# Advances in Primary Mitochondrial Diseases: Diagnosis, Natural History Studies and Clinical Trials

**DOI:** 10.3390/genes17080850

**Published:** 2026-07-23

**Authors:** Albert Z. Lim, Aye-Myat Moe, Renae J. Stefanetti, Yi Shiau Ng

**Affiliations:** 1 Department of Paediatric Neurology, Great North Children’s Hospital, Newcastle upon Tyne Hospitals NHS Foundation Trust, Newcastle upon Tyne NE1 4LP, UK; 2 NHS Highly Specialised Service for Rare Mitochondrial Disorders, Newcastle upon Tyne Hospitals NHS Foundation Trust, Newcastle upon Tyne NE1 4LP, UK; 3 Directorate of Neurosciences, Royal Victoria Infirmary, Newcastle upon Tyne Hospitals NHS Foundation Trust, Newcastle upon Tyne NE1 4LP, UK; 4 Translational and Clinical Research Institute, Newcastle University, Newcastle upon Tyne NE2 4HH, UK; 5 NIHR Newcastle Biomedical Research Centre (BRC), Newcastle upon Tyne NE2 4HH, UK

**Keywords:** whole-genome sequencing, fluid biomarkers, phenotypes, outcome measures, trial endpoints

## Abstract

Primary mitochondrial diseases (PMDs) are one of the most common genetic disorders with an estimated prevalence of 1 in 4300. This review article summarises the latest updates in the field of mitochondrial medicine over the last decade. The availability of exome and genome sequencing in clinical practice has empowered clinicians to unravel the phenotypic heterogeneity of PMD and to end the diagnostic odyssey experienced by many patients and families. In unresolved cases, the detection of variant(s) of unknown significance by next-generation sequencing creates diagnostic and clinical uncertainties, and integrating a multi-omics approach can improve diagnostic yield. Alongside breakthroughs in genomic technologies, there is growing interest in using fluid biomarkers to guide diagnosis, monitor disease progression, and potentially serve as clinical trial endpoints. However, the clinical application of these fluid biomarkers in unselected patient cohorts with different disease onset and phenotypes would require more robust evidence. Natural history studies derived from national and international collaborations have provided insights into genotype–phenotype relationships and prognostic factors across several genotypes, including m.3243A>G, *MT-ATP6*, *POLG*, and *TK2*. Advances in therapeutic discoveries and clinical trials are challenging the obsolete dogma that PMDs are untreatable and bringing hope to patients; four compounds have been licensed, and many trials are in progress. Many barriers and challenges to translating laboratory discoveries into clinical therapy in PMD remain, including preclinical models for efficacy and safety testing, sample size, trial design, and the selection of outcome measures and trial endpoints.

## 1. Introduction

Primary mitochondrial diseases (PMDs) are complex genetic conditions that pose diagnostic and management challenges for clinicians across different disciplines. Many patients manifest with neurological and muscular features during the disease course, and involvement of other high-energy demand organs, such as the heart, kidneys and liver, is frequently evident depending on the underlying genetic causes [1,2]. Specific syndromes, based on constellations of clinical features with multi-system involvement, have been described in PMD to aid prompt recognition and diagnosis [3]. Some genetic variants are associated with different syndromes; for example, the most prevalent mtDNA variant, m.3243A>G, is associated with mitochondrial encephalomyopathy, stroke-like episodes, lactic acidosis (MELAS) syndrome and maternally inherited diabetes and deafness (MIDD). On the other hand, some syndromes can have diverse genetic aetiologies, with Leigh syndrome as a prime example [4]. However, it is well-recognised that many patients with PMD do not fit neatly with syndromic classification, as illustrated by findings of national patient registries [5,6]. Moreover, some patients have an isolated clinical presentation, such as Leber hereditary optic neuropathy (LHON), sensorineural hearing loss, and cardiomyopathy. Establishing a precise genetic diagnosis of PMD has historically been an arduous undertaking, given that mitochondria are controlled by both mitochondrial and nuclear genomes [3]. In addition, management approaches are largely symptomatic and supportive for most patients living with PMD, and disease-modifying treatment is lacking. Nevertheless, establishing a genetic cause is crucial for ending the diagnostic odyssey experienced by patients and their families, fundamental for both genetic and prognostic counselling, and opens the door to research participation [2].

**Figure 1:**
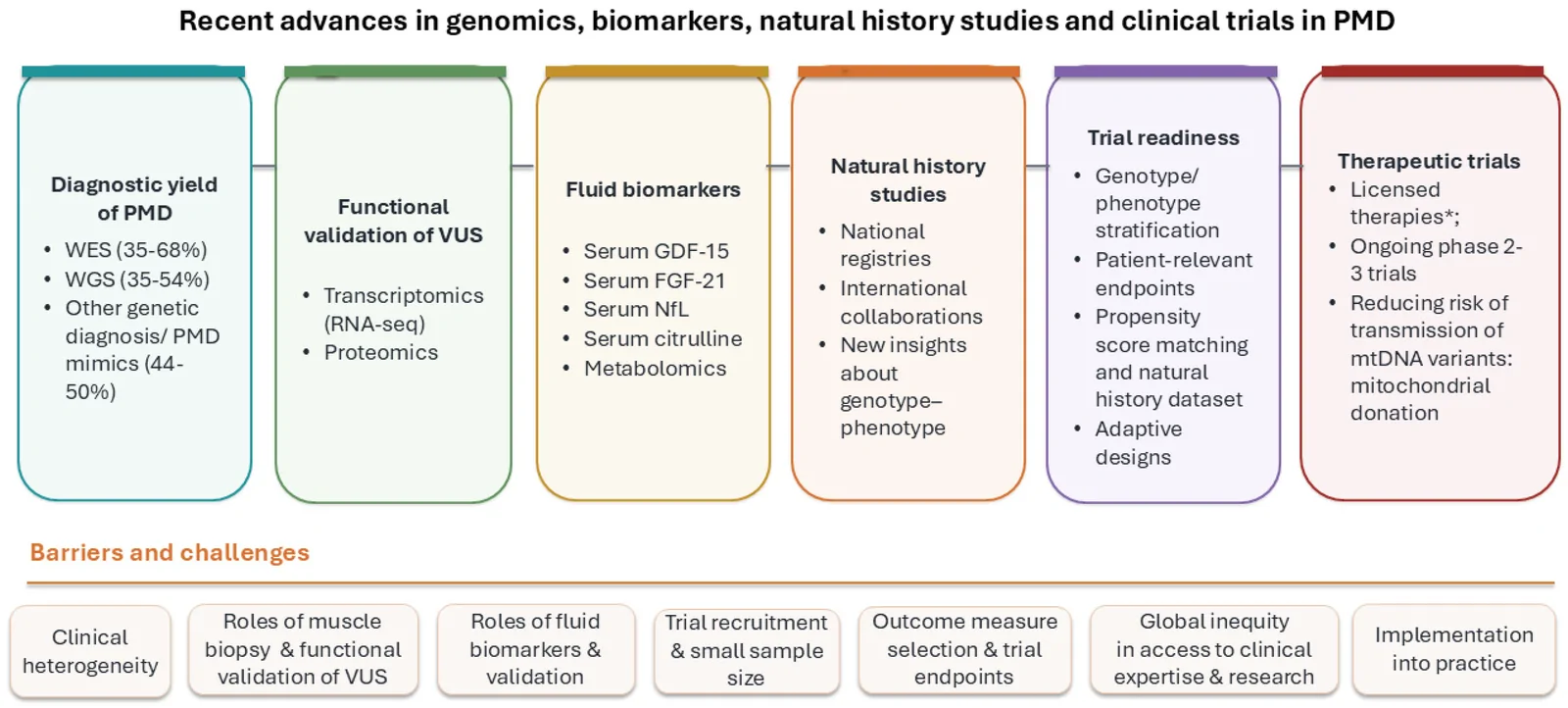
Primary mitochondrial disease: from diagnosis to trial-ready cohorts and emerging therapies. Overview of recent advances in primary mitochondrial disease. Schematic summary of the key themes and their implications for patient stratification and future clinical trial design. * Licensed therapies include idebenone, omaveloxolone, elamipretide and deoxynucleosides. Abbreviations: FGF-21, fibroblast growth factor-21; GDF-15, growth differentiation factor-15; mtDNA, mitochondrial DNA; NfL, neurofilament light chain; PMD, primary mitochondrial disease; VUS, variants of uncertain significance; WES, whole-exome sequencing; WGS, whole-genome sequencing.

Improvements in the diagnostic yield of PMD through exome, genome, and RNA sequencing have been pivotal in raising the profile of these rare diseases in clinical practice over the past decade. The unmet needs for effective treatment in PMD are addressed by national and international coordinated endeavours to set care standards [4], identify therapeutic strategies and outcome measures [5,6], and more recently, conduct multi-centre clinical trials [7]. Whilst there is a plethora of preclinical therapeutic discoveries, challenges in collating robust natural history data across different subtypes of PMD and in identifying responsive biomarkers and outcome measures remain enormous for trial readiness. This narrative review highlights major developments in mitochondrial medicine in the last decade (Figure 1).

## 2. Updates on Diagnostic Approach in Primary Mitochondrial Disease

Primary mitochondrial disease can result from pathogenic variants in both nuclear and mitochondrial genomes. MitoCarta 3.0 provides an updated inventory of mitochondrial proteins, cataloguing more than 1100 proteins with sub-mitochondrial localisation and functional pathways [8]. Establishing a precise genetic diagnosis of PMD is challenging due to multisystem involvement, variable disease onset, overlapping clinical presentation with other genetic and acquired conditions, and a lack of specific biomarkers in clinical practice. Revised classification and new nosology for PMD have been developed to address the complexity of the ever-expanding list of genetic defects in recent years [9,10].

The implementation of targeted gene panels and exome and genome sequencing has driven a paradigm shift from phenotype-first/biopsy-first to a genotype-first investigation strategy for suspected PMD cases [11]. Over two-thirds of adult-onset PMDs are caused by pathogenic mitochondrial DNA variants [12]. Therefore, in adults with suspected PMD, screening for common mtDNA variants in those patients manifesting with classic syndromes or full mitochondrial genome sequencing should be prioritised in clinical settings [2]. The challenge remains in patients with non-specific phenotypes where PMD is one of several differential diagnoses. Similarly, diagnosis is often more complex in infants and young children, where nuclear gene defects are more common [13].

Whole-exome sequencing (WES) transformed the diagnostic yield of PMD between 2010 and 2020, with yields ranging from 35% to 70%, and candidate genes were identified in 24% to 31% of cases [13]. Whole-genome sequencing (WGS) is the next diagnostic tool to consider if the index of genetic disease remains high despite uninformative WES results (Table 1). WGS sequences the entire DNA blueprint, including nuclear and mitochondrial genomes, and captures non-coding regions such as deep intronic variants, with superior performance for detecting structural variant detection compared to WES. Confirmation of PMD using WGS may also nullify the need for the muscle biopsy, which is an invasive procedure that requires general anaesthesia for children. WGS was performed in 345 patients with suspected PMD in the UK 100,000 Genome Project, for whom mtDNA and common nuclear gene causes had already been excluded; 31% of them received a definite or probable genetic diagnosis [14]. As the cost of WGS has decreased and analytic pipelines have advanced over the past few years, it is becoming more cost-effective to apply WGS as a first-tier investigation. The overall diagnostic yield of WGS was ~54% in an Australian cohort comprised of adult patients recruited by a single specialist clinic in Sydney and met the clinical PMD (Nijmegen) criteria; mtDNA variants accounted for 56% of genetic diagnosis [15]. The Australian Genomics Mitochondrial Flagship research programme covering different states also reported similar positive results with WGS (55%), and a higher diagnostic rate was observed in paediatric-onset than adult-onset cases (71% vs. 31%) [16]. Interestingly, a considerably lower diagnostic yield was reported (20%) in a prospective audit of publicly funded WGS over a period of 19 months in Australia [17].

The diagnostic power of WGS can be further exemplified in the acute medical setting of critically ill children, where rapid return of genetic diagnosis can better inform clinical decisions [35,36]. For example, the GEMINI study performed rapid WGS in 400 hospitalised infants and found a significantly higher diagnostic rate compared to targeted gene panels (49% vs. 27%) with a slightly longer median time to result (6.1 days vs. 4.2 days); the molecular diagnosis led to a change of clinical management in nearly 20% of cases [37]. Negative results from WGS should also prompt clinicians to reassess their initial diagnostic formulation and to explore other non-genetic possibilities. If suspicions remain, periodic reanalysis is recommended [38]. Extraction and analysis of mitochondrial DNA data from WES and WGS in genetically undiagnosed patient cohorts led to the identification of pathogenic mtDNA variants in 0.2–0.4% of cases [39,40].

Short-read whole-genome sequencing (srWGS) is widely used in current standard rare disease pipelines, but it faces significant limitations in capturing the full genetic architecture of PMDs and other conditions [41]. srWGS excels at calling single-nucleotide variants, but it can misinterpret low-heteroplasmy mtDNA variants and frequently misses large structural variants and short tandem repeat expansions [41]. Indeed, nuclear encoded mitochondrial segments could be misinterpreted as paternally inherited mtDNA by srWGS in some cases [42]. The use of long-read sequencing technologies, such as Pacific Biosciences Sequencing and Oxford Nanopore Technologies, can overcome the major shortcomings of srWGS and further improve the diagnostic yield [43,44]. For example, the utilisation of long-read sequencing technology in 114 previously undiagnosed families in Europe led to the identification of disease-causing variants in ~12% of these families [45].

A major challenge regularly encountered in the clinical setting is the number of variants of uncertain significance (VUSs) (particularly missense, synonymous and non-coding variants) detected by WES and WGS, in that the uncertainty adds complexity to decision making for clinical management as well as has an adverse psychological impact on patients and their families [46,47]. The role of muscle biopsy has evolved in the era of genomic medicine [11,13,48], and it is considered as a second-line investigation when genetic analysis of blood DNA is inconclusive, functional characterisation of VUS is required, and tissue-specific mtDNA variants such as single, large-scale mtDNA deletion are suspected [27,49].

The integration of genomics, transcriptomics (RNA sequencing) and quantitative proteomics in the diagnostic pipeline, commonly referred to as multi-omics approaches, enables functional characterisation of novel genes and VUSs, thereby increasing the diagnostic yield of rare diseases, including PMDs [11,13]. The Australian Acute Care Genomics programme successfully applied integrated multi-omics to rare disease diagnosis in critically ill infants and children, achieving an overall diagnosis yield of 54% [36]. Other studies have shown that RNA sequencing can contribute to the diagnostic uplift between 10 and 25% in PMD [34,50]. Mass spectrometry-based proteomics, including quantitative assessment of the relative complex abundance of OXPHOS complexes, is emerging as a valuable tool for functional validation of variant pathogenicity and has the potential to replace mitochondrial respiratory chain enzymology testing [51]. However, some mtDNA-encoded heteroplasmic pathogenic variants may only result in an OXPHOS enzymatic defect with normal proteomic analysis.

Although genomics and other -omics technologies can increase diagnostic yield through variant prioritisation and validation, implementation in routine diagnostic services remains constrained by cost, infrastructure, clinical and bioinformatic expertise, interpretative standards and tissue availability. Around 330 million people with rare diseases live in low- and middle-income countries (LMICs), and they have poor access to dedicated clinical services for rare diseases as well as genomic sequencing technologies [52]. There are many initiatives and collaborations involving multiple stakeholders, including governments, academic institutions, industries and international health organisations, to improve equitable access to and delivery of genomic medicine in Africa and other LMICs [53,54,55].

## 3. Updates on Fluid Biomarkers for Primary Mitochondrial Disease

Conventional fluid biomarkers such as serum creatine kinase, serum lactate, and the CSF pyruvate/lactate ratio lack sensitivity and specificity for diagnosing and excluding PMD in clinical practice [56]. Consequently, the diagnostic odyssey has been a recurrent challenge encountered by clinicians and many patients, particularly those presenting with non-syndromic presentations [57].

Fibroblast Growth Factor-21 (FGF-21) is a peptide hormone synthesised by several organs such as the liver, adipose tissue, and muscle that is involved in the regulation of lipid and glucose metabolism [58]. Serum FGF-21 was first shown to correlate with the disease severity of muscle manifesting PMDs and respiratory chain deficient muscle fibres over a decade ago [59] (Table 2). Subsequently, serum growth differentiation factor 15 (GDF-15), one of the transforming growth factor beta family proteins, was reported to be a specific biomarker for PMDs with CNS manifestation [60]. A meta-analysis of diagnostic accuracy for GDF-15 and FGF-21 revealed that serum GDF-15 appeared to have higher sensitivity (83% vs. 71%) and specificity (92% vs. 88%) than serum FGF-21 for discriminating between patients with PMDs and healthy controls [61]. A retrospective study at a tertiary care hospital showed that GDF15 elevation increased the odds of identifying specific subtypes of mitochondrial disorders by 14-fold, including mitochondrial-encoded tRNA variants and mtDNA deletions and depletions (including single, large-scale mtDNA deletions and mtDNA maintenance nuclear genes) [62]. Exercise intolerance and fatigue are common symptoms shared by individuals with chronic fatigue syndrome (CFS) and mitochondrial myopathy. A recent study suggested that serum GDF15 could accurately differentiate patients with PMD from those with CFS, with an area under the curve >0.92, potentially mitigating the unnecessary investigations such as genomic testing and muscle biopsy in those with a normal serum GDF15 level [63]. Studies combining measurements of serum GDF15 and FGF21 have reported mixed findings regarding the ability to predict the final diagnosis of PMD. In the adult mitochondrial disease cohort, combining the two biomarkers did not significantly improve the area under the curve over GDF-15 alone [64]. On the other hand, measurement of both biomarkers has been proposed as a first-line diagnostic investigation for children and adults with suspected mitochondrial disease, as elevated biomarkers were evident in 62% of genetically confirmed mitochondrial disease compared to muscle biopsy abnormalities, which were only present in 39% of cases with genetic diagnosis [65]. The wider availability and reduction in cost of genomic testing may limit the diagnostic application of these serum biomarkers in routine clinical practice. Moreover, these serum biomarkers are elevated moderately in conditions associated with secondary mitochondrial dysfunction, such as liver disease, cardiac failure, and sepsis, as well as other inherited metabolic disorders [66].

In a cross-sectional setting of patients manifesting with myopathy, serum GDF15 level showed small-to-moderate correlations with the six-minute walk test (functional assessment), muscle strength (MRC scale), serum lactate and muscle mtDNA heteroplasmy [75]. However, the monitoring and prognostic values of serum GDF15 and FGF21 have not been firmly established in PMD due to limited longitudinal cohort studies with mixed genotypes [70]. In a study of patients with the pathogenic m.3243A>G variant, GDF15 correlated with the disease burden at the baseline but was not sensitive to detect changes within two years of follow-up [72]. On the other hand, adult patients with TK2 deficiency showed a significant increase in serum GDF15 over time, and a longitudinal study demonstrated serum GDF15 as a potential biomarker for therapeutic response in patients treated with oral deoxynucleotides [76].

Whilst stress-induced plasma GDF15 reactivity appeared blunted, saliva GDF15 showed response to experimental laboratory mental stress in patients with PMD compared to controls. Good correlations between saliva GDF15 and neurological symptoms, fatigue and functional capacity have also been observed [77]. However, the effect size was smaller in saliva GDF15 compared to the blood level.

Neurofilament light chains (NfLs) are subunits of neurofilament proteins that are in the cytoplasm of large, myelinated axons. A raised serum NfL level reflects neuronal injury, and it correlates with the cerebrospinal fluid NfL level in several common neurological disorders such as multiple sclerosis, motor neurone disease, immune-mediated peripheral neuropathies, and neurodegenerative diseases [78,79]. More recently, a raised serum NfL level has been identified in PMDs with CNS involvement, including those manifesting with mitochondrial encephalomyopathy, lactic acidosis and stroke-like episodes (MELAS) syndrome and epilepsy, as well as during acute stroke-like episodes [80,81,82]. Correlations of serum NfL levels, cognitive performance (measured by Mini-Mental State Examination), and the volume of metabolic stroke lesions have been reported [81]. Interestingly, plasma NfL level was elevated in patients with POLG-related mitochondrial disease, irrespective of clinical phenotypes: the levels were not significantly different between the ataxia neuropathy spectrum and myopathic phenotype (including chronic progressive external ophthalmoplegia) [83]. Serum NfL appears to be a promising fluid biomarker for episodic crises affecting brain and neurodegenerative processes in PMDs. However, larger prospective cohort studies are crucial to delineate the evolution of serum NfL levels at different stages of PMDs and potentially as an exploratory clinical trial endpoint.

The association of low citrulline levels and several subtypes of PMDs, including Pearson syndrome, LS and MELAS syndrome, has been reported since the 1990s [66,84]. A prospective genetic diagnosis of *MT-ATP6* has been made in presymptomatic babies with hypocitrullinaemia identified by the newborn screening programme in the US [73]. Hypocitrullinaemia likely reflects impaired ATP-dependent citrulline synthesis within enterocyte and hepatic mitochondria, particularly involving carbamoyl phosphate synthetase 1 (CPS1), a key mitochondrial urea cycle enzyme. This appears especially relevant in MT-ATP6-related disease, where complex V deficiency impairs ATP production and may lead to secondary CPS1 dysfunction and reduced citrulline synthesis. Adult-onset MT-ATP6-related disease appears to have normal citrulline levels, unlike patients with infantile and childhood manifestations, suggesting its predictive value for disease onset and severity [85]. Further prospective studies are required to validate plasma citrulline as a prognostic and monitoring biomarker in clinical and trial settings.

Metabolomics studies have flourished in clinical medicine, given their potential to improve the understanding of pathological mechanisms and to identify biomarkers for diagnosis, disease progression monitoring, and prognosis [86]. Initial metabolomics research studies in mitochondrial disease focused on cell and animal models [87]. More recently, human studies recruiting different patient cohorts have generated interesting insights [88], such as novel discovery of metabolites (N-lactoyl-amino acids, β-hydroxy acylcarnitines, and β-hydroxy fatty acids) that showed good correlation with disease severity in patients with m.3243A>G-related mitochondrial disease [89] and disease-specific biomarker profiles in different subtypes of PMD and inclusion body myositis with secondary mitochondrial dysfunction [90].

## 4. Updates on the Natural History Studies

**Figure 2:**
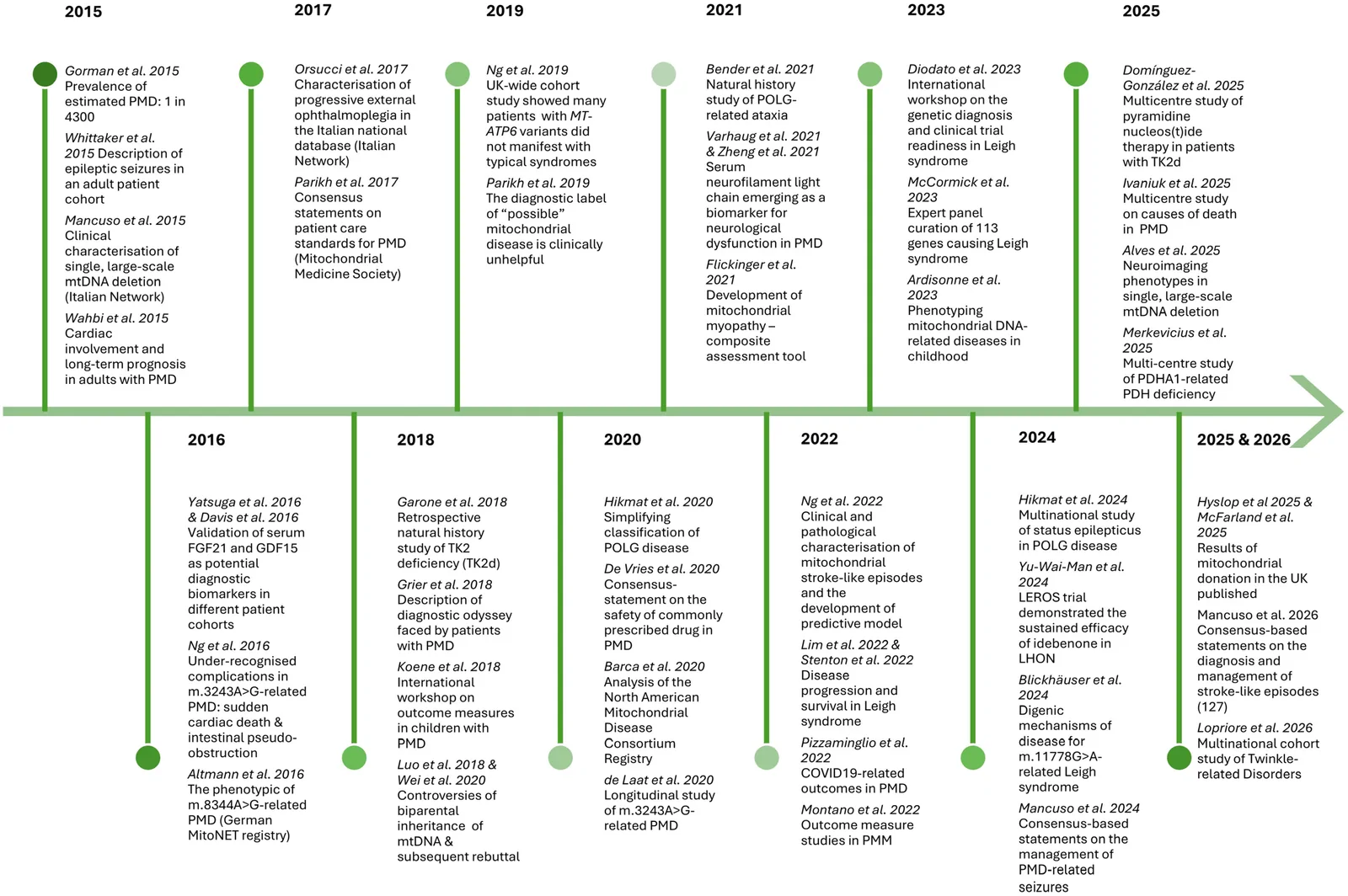
Selected publications related to clinical research and natural history studies in PMD between 2015 and June 2026 [4,6,12,42,60,64,80,81,93,99,100,101,102,103,104,105,106,107,108,109,110,111,112,113,114,115,116,117,118,119,120,121,122,123,124,125,126,127,128,129,130,131,132,133,134]. LHON, Leber hereditary optic neuropathy; PMD, primary mitochondrial disease; PMM, primary mitochondrial myopathy.

Natural history studies are essential for uncovering disease progression and prognostic factors that can inform better clinical management and better clinical trial designs (Figure 2). Several cohort studies have shown that most patients harbouring the m.3243A>G variant in the *MT-TL1* gene, the most prevalent pathogenic mtDNA variant in the population, develop diabetes mellitus and sensorineural hearing loss (SNHL), and less than a quarter of patients develop devastating stroke-like episodes and seizures [91,92]. A recent multi-centre longitudinal natural history study has taken the cohort results further to develop a prediction model for the risk of stroke-like episodes in carriers of the pathogenic m.3243A>G variant by incorporating four independent variables: age-corrected blood heteroplasmy, resting plasma lactate level, body mass index and severity of sensorineural hearing loss [93]. There are additional inherent challenges in understanding the natural history of mitochondrial DNA disorders, as the trajectory of disease progression is confounded by unique characteristics of mtDNA genetics such as heteroplasmy, the threshold effect, genetic bottlenecks, tissue specificity [94,95,96], and other genetic modifiers [97] and environmental factors [98]. The manifestation of dystonia and Leigh-like neuroimaging changes without optic neuropathy among carriers of primary mtDNA variants associated with LHON, including m.11778G>A, has been enigmatic, and the discovery of digenic co-occurrence of nuclear-encoded heterozygous pathogenic variants of the complex I subunit provides a plausible mechanistic explanation [99].

Longitudinal cohort studies encompassing patients with a broad spectrum of genotypes and phenotypes challenge the dogma that childhood-onset PMD is associated with uniformly high morbidity and mortality. Leigh syndrome (LS), a progressive neurodegenerative mitochondrial disorder, historically had a reported mean survival of under two years in the early literature [135]. Since the first described postmortem neuropathological entity in 1951 [136], the genotypic spectrum has expanded substantially with genetic defects in 16 mitochondrial-encoded genes and approaching 100 nuclear genes associated with LS [123]. Many children with LS are surviving the regressive crises and living longer with better medical care; cases of adult-onset LS are also emerging [111,121,137,138]. Natural history studies in this genetically heterogeneous condition have also identified factors that could influence disease burden and progression over time [139]. One common theme across large cohorts from the UK [120], China [121] and Italy [140] is the unfavourable outcomes among those who harbour pathogenic *SURF1* variants. Other genetic variants appeared to have a better prognosis, with some living to adulthood [141]. This insight from natural history studies also highlights the importance of confirming a molecular genetic basis in PMD syndromes to allow for better prognostication and counselling.

Our knowledge on the phenotypic spectrum and disease severity of different PMDs is constantly expanding with datasets that provide longitudinal views, highlighting the earlier literature, which was often biased towards those with syndromic presentations and severe disease burden. A recent multi-centre retrospective study has highlighted that many patients with POLG disease develop a continuum of clinical features over time, rather than discrete syndromes proposed in the early literature [113]. Such insights have simplified the classification of POLG-related disease, which was previously complicated by incongruent syndromic categorisations. Early disease onset, significant hepatic dysfunction and status epilepticus are poor prognostic factors in patients with POLG disease [125,142,143]. Ataxia is a common manifestation of POLG-related mitochondrial disease [144,145,146], and a prospective natural history study has demonstrated that age of onset influences disease progression, suggesting that the Scale for the Assessment and Rating of Ataxia (SARA) is a suitable primary trial endpoint [118].

Longitudinal cohort data are also redefining pyruvate dehydrogenase complex deficiency (PDCD) as part of the broader spectrum of mitochondrial disease rather than a disorder of intermediary metabolism. These studies have demonstrated the marked phenotypic and genetic heterogeneity of PDCD, ranging from severe neonatal encephalopathy and congenital lactic acidosis to milder childhood or adult-onset presentations with intermittent ataxia, neuropathy or LS [147]. A large recent international multi-centre cohort of 891 individuals with PDHA1-related disease (X-linked) demonstrated marked phenotypic and genetic heterogeneity, ranging from severe neonatal encephalopathy and congenital brain malformations to intermittent ataxia and adult-onset presentations [130]. Disease severity and survival were influenced by variant type, sex and age at onset, while female carriers were increasingly recognised to manifest clinically significant disease, including stroke-like episodes, peripheral neuropathy and Leigh-like imaging abnormalities [148]. Registry studies from the North American Mitochondrial Disease Consortium identified PDCD as the second most common genetically resolved mitochondrial disorder after POLG-related disease [115]; clinicians need to gain familiarity with managing this condition, including ketogenic diet and treatment of congenital lactic acidosis [149].

To address the deficit in our understanding of the natural history of mitochondrial disorders, several national cohort studies of PMDs have been established over the past two decades [92,105,150,151,152]. Beyond national registries, international collaboration by the European Network for Mitochondrial diseases (GENOMIT) has recruited over 2000 paediatric patients and developed open-access resources to delineate genotype–phenotype associations [153]. A better understanding of natural history and disease progression across different types of PMDs is vital for informing the design of clinical trials as well as selecting appropriate outcome measures and trial endpoints. Given the constraints of sample size and the ethical dilemma of having placebo arms in rare disease trials, several clinical trials, such as omaveloxolone in Friedreich’s ataxia [154] and idebenone in LHON [126], have compared the treated trial cohorts against the untreated natural history registries using propensity score matching.

There is growing interest among patient advocacy and charity groups in engaging with natural history studies. The Leigh Syndrome International Consortium has been established by several key stakeholders that include national patient organisations from Europe, North America and Australia with the aim of establishing best practices from PMD studies [155]. Moreover, longitudinal tracking of patient experience through electronic capture of patient-reported outcome measures, including quality of life, fatigue, and functional performance, will complement the clinical observation of disease progression and evaluation of targeted therapeutic effects [156].

Longitudinal observations in natural history studies have shed light on certain trends beyond the traditional neurological manifestations of PMD. Some have challenged the prevailing dogma of generic screening for cardiac manifestation in PMDs and proposed a more targeted approach in adult patients with high-risk genotypes [157,158,159]. Therefore, specific PMD patients with negligible risk of developing a cardiac disease can be reassured without requiring universal screening, potentially reducing healthcare costs. Management of patients who develop progressive organ failure frequently imposes challenges and dilemmas in clinical practice, given concerns about multisystem disease and survival and the scarcity of donated organs. Growing evidence has suggested favourable long-term outcomes in carefully selected patients with PMD receiving kidney and heart transplantations, although they might be at risk of developing metabolic decompensation related to mitochondrial dysfunction, neurological deterioration and other peri-operative complications [160,161,162]. The outcomes of liver transplantation are more heterogeneous in mtDNA depletion syndrome caused by *POLG* and *DGUOK* variants due to underlying severe CNS disease, although long survival has been observed in *MPV17* cases [163,164].

A systematic review of natural history studies in mitochondrial disorders showed that the majority of studies were based on genotype rather than clinical phenotype and that a multi-centre collaborative approach is key to a better definition of disease progression and developing targeted treatments [165]. One of the collaborations that underpins this point is the unique practice model among clinicians in Spain for identifying the natural history TK2 deficiency in both adults and children [166,167,168]. This has been partly driven by the potential effective treatments for TK2 deficiency using deoxynucleosides.

## 5. Updates on Treatments and Therapeutic Trials for Primary Mitochondrial Disease

The use of vitamins and cofactors is widely recommended by many clinicians treating children and adults affected by PMDs, although there are no robust and demonstrable benefits in most types of PMDs. However, prescribing differences in the use of individual vitamins and cofactors are related to region (North America vs. Europe) and clinical specialty (paediatrics vs. adult medicine) [169]. Similarly, administration of intravenous L-arginine has been historically advocated for managing stroke-like episodes in some clinical settings, even though the evidence supporting its use has been questioned [170]. To streamline the acute management of MELAS syndrome, international consensus statements have been produced by key experts in the field, and the use of L-arginine in both acute and chronic settings has not been recommended due to its unproven efficacy [131,171].

Idebenone is a benzoquinone thought to facilitate electron transfer from complex I to complex III and restore ATP production. The use of idebenone for patients with LHON in Europe was approved in 2015. A multi-centre, retrospective, open-label study of long-term treatment with idebenone showed that 53% of patients had a clinically relevant recovery of vision, with a mean treatment duration of 23.8 months [172]. Using the external, untreated natural history data as a comparator, the LEROS study provided further evidence of the sustained efficacy of idebenone in patients with LHON, with a clinically relevant benefit observed in 42% of patients at 12 months and 53% at 24 months.

Another recent progress in LHON is allotopic gene therapy in the form of intravitreal injection of the recombinant adeno-associated virus 2 (rAVV2/2) vector encoding the human wild-type ND4 gene. Two phase 3, randomised, double blind, sham-controlled studies, RESCUE (*n* = 39) and REVERSE (*n* = 37), enrolled adult patients with the pathogenic m.11778G>A variant in *MT-ND4* with visual loss of six months or less and six to twelve months, respectively. Patients were followed up for 96 weeks, and improvement in best-corrected visual acuity (BCVA) in both eyes was observed but did not meet the primary endpoint [173,174]. The RESTORE trial, a 5-year follow-up study, demonstrated sustained bilateral improvement in BCVA and a good safety profile [175]. A recent study, REFLECT, reported greater improvement in BCVA when treatment was given to both eyes compared to one eye [176]. However, the mechanism underlying the unexpected bilateral visual improvement in unilaterally treated eyes remains elusive [173], and regulatory stakeholders recommend a further RCT before seeking authorisation.

Fatigue is a debilitating symptom frequently reported by patients with PMDs across different genotypes. The exact mechanisms of fatigue related to mitochondrial disease remain elusive but are likely multifactorial, including perceived fatigue [177], muscle fatigue due to exercise intolerance [178], and other medical comorbidities. Some endeavours have been taken to measure and characterise fatigue clinically [177,179,180], and different trajectories of fatigue level have been observed in a recent two-year follow up study [181]. Importantly, subjective fatigue and patient-reported outcomes may not always align with objective measures of disease burden, functional capacity or real-world physical activity [182]. This discrepancy highlights the complexity of interpreting symptomatic change in therapeutic trials, particularly when interventions aim to improve fatigue, exercise tolerance or daily functioning. Amelioration of symptoms related to primary mitochondrial myopathy, including fatigue, has been targeted by several small-molecule drugs, such as omaveloxolone and resveratrol, and repurposed drugs, such as bezafibrate and acipimox, over the last decade [183] (Table 3).

Omaveloxolone is a semi-synthetic oleanolic triterpenoid that activates the nuclear factor erythroid-derived 2-related factor 2 (Nrf2) pathway, which plays a crucial role in cellular resistance to oxidative stress [211]. A randomised, double-blind phase 3 trial of omaveloxolone did not demonstrate any improvement in peak cycling exercise workload (primary endpoint) and six-minute walk test (6MWT, secondary endpoint) at 12 weeks but appeared to be well-tolerated by patients with mitochondrial myopathy [202]. On the other hand, omaveloxolone has recently been approved by the FDA (US) and EMA (EU) for the treatment of adult patients with Friedreich’s ataxia based on the positive results from the placebo-controlled MOXIe Part 2 trial and the open-label extension study [203,212].

Elamipretide is a mitochondrial cardiolipin binder that was shown to be safe and potentially efficacious in improving muscle function in adult patients with primary mitochondrial myopathy in a phase 2 RCT [189]. However, the efficacy of elamipretide was not demonstrated in the subsequent multi-centre, phase 3 trial that recruited an impressive number of patients (n = 218) [190]. Elamipretide has also been trialled in Barth syndrome, an ultra-rare and severe X-linked disorder of mitochondrial cardiolipin metabolism typically manifesting with infantile-onset cardiomyopathy, skeletal myopathy, growth delay and other features. The results from the RCT (TAZPOWER) and open-label study demonstrated sustained tolerability and safety, as well as improvements in functional assessments (including the 6MWT) and cardiac function [191], paving the way for FDA approval in 2025.

Sonlicromnanol (KH176) is a reductive and oxidative distress modulator that selectively inhibits microsomal prostaglandin E1 synthase activity. In a phase 2b trial involving 27 patients with m.3243A>G-related mitochondrial disease, sonlicromanol was well tolerated and safe, although there was no significant change in the primary endpoint, the attentional domain of the cognitive score. Other secondary outcome measures, including quality-of-life questionnaires, showed clinically meaningful improvements [208]. Currently, a multi-centre phase 3 trial is recruiting participants.

There are also clinical trials that are focused on specific genetic diagnoses. In an open-label study investigating the compassionate use of deoxynucleosides (MT-1621) in patients with TK2 deficiency compared to untreated external cases identified from the literature, the most striking findings were increase in survival (death rate 0% vs. 58%), regained lost motor milestones (65% vs. 4%), decreased ventilatory support duration in 28% of treated patients, and feeding support being discontinued in three patients [127]. Based on the safety profile and encouraging results from the open-label study, the FDA and EMA approved MT-1621 for children and adults with disease onset ≤ 12 years, in Nov 2025 and March 2026, respectively.

The progress of therapeutic development in Leigh syndrome has been limited to preclinical discoveries, such as therapeutic hypoxia [213,214], over the last decade. Vatiquinone, formerly known as EPI-743, has been trialled in LS and mitochondrial epileptic encephalopathy. However, the robustness of the trial findings was questionable due to the study design and selection of trial endpoints [215]. More recently, a pluripotent stem-cell-based drug screening approach identified sildenafil, a PDE5 inhibitor, which extended life span of LS animal models and improved symptoms in off-label use in six patients with pathogenic *MT-ATP6* variants [216]. However, as an open-label compassionate use case series without a randomised, placebo-controlled arm, this study with such a small sample size is prone to investigator bias, and a firm conclusion cannot be drawn due to natural fluctuations in the disease trajectory in PMD. Thus, a properly designed RCT with clearly defined trial endpoints is imperative to demonstrate the safety and efficacy of sildenafil in patients with LS and other clinical phenotypes.

Pearson syndrome is a devastating mitochondrial disorder in children, which is characterised by sideroblastic anaemia and pancreatic insufficiency with poor outcomes resulting in early death in childhood. Mitochondrial augmentation therapy (MAT), a technology that enriches patient-derived CD34+ hematopoietic stem and progenitor cells with wild-type mitochondria before infusion, was first reported in 2018 [217]. This was followed by an early-phase clinical trial recruiting patients with single, large-scale mitochondrial DNA (mtDNA) deletion syndromes, including Kearns–Sayre syndrome. Interestingly, MAT was associated with increased peripheral blood mtDNA copy number following treatment, suggesting that transplantation of wild-type mitochondria may improve mitochondrial biogenesis or selectively expand haematopoietic cells with a lower burden of pathogenic mtDNA deletions. This observation is particularly interesting because mtDNA depletion and the clonal expansion of deleted mtDNA molecules are thought to contribute to disease progression in mitochondrial deletion syndromes. The investigators also noted improvement in aerobic function, an increase in body weight, and an increase in quality-of-life measurements [218].

The onset and severity of mitochondrial DNA disease can be influenced by the heteroplasmic state and threshold effect of individual mtDNA variants, such as m.8344A>G in *MT-TK* and many pathogenic *MT-ATP6* variants [111,219]. Some homoplasmic variants, such as three common LHON mtDNA variants, exhibit reduced clinical penetrance and sex dimorphism, with male carriers being at higher risk of visual loss [220]. In the absence of effective disease-modifying therapy in mtDNA-related PMD, reproductive options such as prenatal diagnostic testing and pre-implantation genetic testing are reliable measures to reduce the transmission of heteroplasmic mtDNA variants [96,221]. Mitochondrial donation, an IVF-based technique that involves the microsurgical transplantation of the nuclear genome from a carrier egg to an unaffected enucleated egg, offers an alternative means of potentially reducing the risk of mtDNA disease in women who carry (near) homoplasmic pathogenic mtDNA variants [222]. The Newcastle team in the UK recently reported that 36% of women (8/22) became pregnant through an intracytoplasmic sperm injection procedure for pronuclear transfer (PNT) and eight babies were born, with the levels of heteroplasmy for maternal pathogenic mtDNA variant not detectable in five newborns, 5% in one newborn and <20% in two others [132]. All eight neonates were healthy at birth and appeared to have made normal developmental progress at their most recent assessment; medical problems were identified in three children, which included self-limiting myoclonic epilepsy of infancy, an uncomplicated urinary tract infection, and hyperlipidaemia and left dilated ventricle attributed to atrial tachyarrhythmia that both responded to treatments [133]. Whilst the reported outcomes of strictly regulated mitochondrial donation are generally welcome in the patient and scientific communities, there are ongoing ethical and scientific debates, as well as legitimate concerns surrounding the cost effectiveness, the risk of mtDNA heteroplasmy carry-over associated with PNT, and the phenomenon of mtDNA reversal [223,224,225].

Instrumented balance and gait analysis, either in the laboratory or using wearables, is emerging as a promising digital outcome measure that can objectively capture motor and functional impairment, as well as disease progression over a short time frame, in neurological disorders such as Parkinson’s disease, hereditary ataxia and myopathy. Instrumented gait studies in hereditary ataxia have demonstrated their sensitivity to quantify changes when compared to clinician-reported measure such as the ataxia rating scale [226]. A specific gait metric, stride velocity 95th centile, has been accepted as a primary trial endpoint for Duchenne muscular dystrophy by the EMA [227]. However, there have been limited published studies reporting the utility of laboratory gait analysis and accelerometer in children and adults with PMD to date. Similar to other diseases, there are major hurdles and challenges for harnessing digital health technologies into clinically meaningful trial endpoints in PMD; these include marked clinical heterogeneity, harmonisation of study protocols, development of a technical and analytical framework, cross-disciplinary collaboration, and longitudinal data collection.

The move towards more robust clinical trial designs, including greater adoption of randomised control trials, has been a very positive development for evaluating therapeutic compounds in the mitochondrial field and upholding scientific rigour [228]. However, this progress is accompanied by inherent challenges. Recruitment remains a major barrier, contributing to the premature termination of several clinical trials (Table 3). Trial design is further complicated by marked genetic and phenotypic heterogeneity, raising debates about whether to ‘split’ participants into genetically or clinically defined subgroups or ‘lump’ broader patient populations to achieve feasible sample sizes. Recent experiences in primary mitochondrial myopathy trials have also highlighted the difficulties of translating encouraging early-phase signals into successful phase 3 outcomes [200]. In parallel, the psychometric properties of many commonly used outcome measures, including the validity, reliability, responsiveness, and clinical meaningfulness, remain untested or unestablished in patients with PMD, despite expert recommendations. Moreover, it is imperative to consider the effect size of any treatment, the sensitivity and specificity of outcome measures for measuring changes, predefined minimal clinically meaningful changes agreed upon by regulatory stakeholders, operational financial costs, and participant burden when planning a clinical trial. These challenges highlight the importance of integrating patient perspectives and lived experience [229,230] into trial design and endpoint selection, a priority increasingly recognised by stakeholders. Given these challenges, novel trial approaches should also be considered alongside conventional randomised controlled trials. Adaptive, platform, basket or umbrella trial designs may allow multiple therapies [231] and genotypes or phenotypic subgroups to be evaluated more efficiently, particularly when supported by robust natural history data, validated endpoints and international recruitment networks. The multimodal profiling approach used in dementia research, which integrates genetic, imaging, and digital and fluid biomarkers [232], is applicable to the field of PMD and could refine patient stratification and potentially reduce sample size in clinical trials. Moreover, the application of a precision medicine approach, such as using patient-derived cell models to perform personalised pharmacological screening [233], has the appeal of maximising the discovery of efficacious therapies, mitigating the individual risk of toxicity and reducing the futility in future trials in PMD.

Many different gene therapy approaches, including recombinant adeno-associated virus-based gene replacement, transcription activator-like effector nucleases, zinc-finger nucleases, and CRISPR-free gene editing, have shown encouraging findings in preclinical models of PMD [234]. However, there are outstanding challenges to the clinical translation of gene therapy for PMD, such as technical challenges of CNS transduction, safety considerations, trial design, patient recruitment and financial costs.

## 6. Conclusions

The past decade has seen substantial progress in the diagnosis and clinical characterisation of primary mitochondrial diseases, driven by advances in genomic sequencing, multi-omic approaches, biomarker discovery and increasing natural history studies. These developments have improved diagnostic precision, expanded the understanding of genotype–phenotype relationships, and strengthened the foundations required for trial readiness. However, major challenges remain, including unresolved diagnoses, inequal access to clinical expertise and advanced diagnostic facilities, limited validation of biomarkers and outcome measures, and the difficulty of conducting adequately powered trials. Future progress will depend on international collaboration, harmonised longitudinal datasets, meaningful patient involvement, and trial designs that can accommodate biological complexity while generating robust evidence. Together, these approaches offer a realistic path towards earlier diagnosis, better patient management and more effective, targeted therapies for people living with mitochondrial disease.

## Figures and Tables

**Table 1:** Summary of publications reporting on the diagnostic performance of exome sequencing, genome sequencing and other-omics technologies between 2015 and June 2026. Findings from targeted gene panel and WES across different studies conducted between 2010 and June 2020 have been summarised elsewhere [13]. MDC, mitochondrial disease criteria; MNC, Modified Nijmegen Criteria; N/A, not applicable; NMDC, Nijmegen Mitochondrial Disease Criteria. ^a^ Targeted gene panel applied to 125 patients and WES performed in 10 patients; ^b^ 197 out of 303 were WES-inconclusive cases; ^c^ targeted gene panel, WES +/− WGS; ^d^ for the validation cohort analysis (untargeted proteomics approach vs. respiratory chain enzymology), a cohort of 24 patients with confirmed genetic diagnoses based on known pathogenic or likely pathogenic nuclear or mtDNA-encoded variants. ^e^ Randomised to WES + mtDNA or WGS.

Publication	Method	Age Group	Sample Type	*N*	Criteria	Definitive Diagnostic Rate	Proportion of Nuclear PMD Diagnosis	Proportion of mtDNA Diagnosis	Proportion of Non-PMD Diagnosis	Proportion of Candidate Genes
Wortmann et al. [18]	WES	Paediatric & adult	Blood & muscle	109	NMDC	38.5% (42/109)	50% (21/42)	None (already excluded)	50% (21/42)	N/A
Pronicka et al. [19]	WES	Paediatric	Blood & fibroblasts	113	MDC	59.3% (67/113)	62.7% (42/67)	9% (6/67)	28.3% (19/67)	N/A
Kohda et al. [20]	WES & mtDNA	Paediatric	Blood & muscle	142	Other	34.5% (49/142)	65.3% (32/49)	20.4% (10/49)	14.3% (7/49)	37.3% (53/142)
Legati et al. [21]	Panel & WES	Paediatric & adult	Blood & muscle	125	Other	15.2% (19/125); 6/10 (60%) ^a^	15.2% (19/125); 33.3% (2/6)	None (already excluded)	66.7% (4/6)	21.6% (27/125)
Puusepp et al. [22]	WES	Paediatric	Blood & muscle	28	MDC	57% (16/28)	18.9% (3/16)	6.3% (1/16)	75% (12/16)	N/A
Theunissen et al. [23]	mtDNA & WES	Paediatric & adult	Blood	117	MDC	68% (80/117)	45% (36/80)	28.8% (23/80)	26.2% (21/80)	7% (8/117)
Grigalionienė et al. [24]	Sanger, Panel & WES	Paediatric & adult	Blood	83	NMDC	18/83 (21.7%)	55.6% (10/18)	38.9% (7/18)	8.4% (7/83)	N/A
Riley et al. [25]	WGS	Paediatric	Blood	40	NMDC	55% (22/40)	50% (11/22)	18.2% (4/22)	31.8% (7/22)	12.5% (5/40)
Tsang et al. [26]	WES	Paediatric	Blood	66	MDC	35% (23/66)	47.8% (11/23)	N/A	52.2% (12/23)	N/A
Schon et al. [14]	WGS	Paediatric and adult	Blood	345	NMDC	30.7% (98/319)	28.9% (30/98)	3.1% (5/98)	66.3% (65/98)	1.9% (6/319)
Forny et al. [27]	WES	Paediatric	Blood & muscle	85	Other	36.5% (31/85)	67.7% (21/31)	9.7% (3/31)	22.6% (7/31)	N/A
Davis et al. [15]	WGS	Paediatric & adult	Blood	242	NMDC	53.7% (130/242)	38.5% (50/130)	56.2% (73/130)	5.4% (7/130)	4.5% (11/242)
Yépez et al. [28]	WES & RNA-Seq	Paediatric & adult	Blood & fibroblasts	303 ^b^	Other	16% (32/197)	16% (32/197)	90.6% (29/32)	9.4% (3/32)	6.1% (12/197)
Wu et al. [29]	WGS	Paediatric	Blood	503	MDC	35.2% (177/503)	17.5% (31/177)	8.8% (15/177)	74% (131/177)	N/A
Rouzier et al. [30]	Mixed ^c^	Paediatric & adult	Blood & fibroblasts	397	MDC	81% had PMD	81% had PMD	N/A	19%	N/A
Ouyang et al. [31]	WES & mtDNA	Paediatric	Blood	40	Other	45% (18/40)	5.6% (1/18)	5.6% (1/18)	89% (16/18)	N/A
Ambrose et al. [32]	Mixed ^c^	Paediatric & adult	Blood	403	Other	23.1% (93/403)	32.2% (30/93)	44.1% (41/93)	23.7% (22/93)	1.9% (6/310)
Hock et al. [33]	Proteomics	Paediatric & adult	Blood, fibroblasts & muscle	24	MNC	83.3% (20/24) vs. 79.2% (19/24) ^d^	19	5	N/A	N/A
Rius et al. [16]	Mixed ^e^	Paediatric & adult	Blood & muscle	140	MNC	55% (77/140)	48% (37/77)	23.3% (18/77)	28.6% (22/77)	6.4% (9/140)
Ball et al. [17]	WGS	Paediatric & adult	Blood	300	Other	20% (61/300)	16.4% (10/61)	39.3% (24/61)	44.3% (27/61)	21.3% (51/239)
Liu et al. [34]	WES, WGS & RNA-seq	Paediatric	Blood & fibroblasts	140	MDC	25% (35/140)	25% (35/140)	N/A	Not specified	8.6% (12/140)

**Table 2:** Overview of four blood biomarkers for primary mitochondrial disease. MELAS, mitochondrial encephalomyopathy, lactic acidosis and stroke-like episodes; MND, motor neurone disease (also known as amyotrophic lateral sclerosis); MS, multiple sclerosis; NAFLD, non-alcoholic fatty liver disease; PMD, primary mitochondrial disease.

Biomarker	Biological Source	Disease Association	Strengths	Limitations	Current Clinical Utility
FGF-21 (Fibroblast Growth Factor 21)	Skeletal muscle (secreted during mitochondrial stress/UPR^mt^)	PMD with prominent myopathy; pathogenic variants affecting mtDNA translation and maintenance [59,67]	High specificity for mitochondrial myopathies; good correlation with disease severity in paediatric cases [68]	Can be elevated in non-mitochondrial diseases (obesity, NAFLD, type 2 diabetes mellitus, chronic kidney disease, and heart failure)	Screening biomarker to guide genetic investigation [69]; the role as monitoring biomarker not established [70,71]
GDF-15 (Growth Differentiation Factor 15)	Ubiquitous tissue expression; markedly elevated by mitochondrial stress	Broad range of PMDs (myopathy, encephalopathy, multisystemic)	Higher overall diagnostic accuracy compared to FGF-21 [61]	Can be elevated in cardiovascular disease, cancer, inflammation, and advanced age	Screening biomarker to guide genetic investigation; the role as monitoring biomarker not established [71,72]
NfL (Neurofilament Light Chain)	Axonal cytoplasm of central and peripheral neurons	PMD with prominent neurodegenerative features or neuropathy (e.g., MELAS, POLG variants) [73,74,75]	Indicator of active axonal damage and neurodegeneration	Cannot differentiate PMD-related neurodegeneration from other neurological diseases (e.g., MS, MND).	Potentially could be used as monitoring biomarker and surrogate endpoint in clinical trials to monitor neuroprotection
Citrulline	Inhibition of citrulline production in the mitochondrial matrix of enterocytes in the small intestine	MT-ATP6-related PMD	Specificity in MT-ATP6 related PMDs such as Leigh syndrome manifestation has been suggested	Non-specific to PMD; low citrulline level is observed in proximal urea cycle disorder	Could be used as newborn screening biomarker for pathogenic *MT-ATP6* variants [73,74]

**Table 3:** Summary of registered clinical trials in primary mitochondrial disease (as of 25 May 2026). Included studies were those recruiting, completed, or published between 2015 and 2026. Where available, N refers to the published analysis population (e.g., completers or Full Analysis Set [FAS]) or as reported on ClinicalTrials.gov. Abbreviations: 5XSST = Five Times Sit-To-Stand Test; 30sec-STS = 30 Second Sit-to-Stand Test; 6MWT = six-minute walk test; 12MWT = twelve minute walk test; AAV = adeno-associated virus; AEs = adverse events; AESI = TEAE of Special Interest; ATP = adenosine triphosphate; BCVA = best-corrected visual acuity; BTHS-SA = BarTH Syndrome Symptom Assessment; C-SSRS = Columbia-Suicide Severity Rating Scale; DB = double-blind; EE-TP = Erythrocyte Encapsulated Thymidine Phosphorylase; ETDRS = Early Treatment Diabetic Retinopathy Study chart; FAS = Full Analysis Set; GMFM-88 = Gross Motor Function Measure-88; HAMA = Hamilton Anxiety Scale; HAMD = Hamilton Depression Scale; HR = heart rate; intravenous = IV; JMDRS = Japanese mitochondrial disease rating scale; LHON = Leber’s Hereditary Optic Neuropathy; MELAS = mitochondrial encephalopathy lactic acidosis & stroke-like episodes; mFARS = Modified Friedreich’s Ataxia Rating Scale; MMSE = Mini-Mental State Examination; MRS = magnetic resonance spectroscopy; MRSI = magnetic resonance spectroscopic imaging; MNGIE = mitochondrial neurogastrointestinal encephalomyopathy; MRI, magnetic resonance imaging; mtDNA = mitochondrial DNA; NA = not available; nPMD = nuclear DNA primary mitochondrial disease; NPMDS = Newcastle Paediatric Mitochondrial Disease Scale; PedsQL = Paediatric Quality of Life Inventory; PEO = progressive external ophthalmoplegia; PMM = primary mitochondrial myopathy; PMMSA = Primary Mitochondrial Myopathy Symptom Assessment; PROMIS = Patient-Reported Outcome Measurement Information System; rAAV2-ND4 = recombinant Adeno-Associated Virus-NADH dehydrogenase, subunit 4 (complex I); SAE = serious adverse events; SF = short form; TEAEs = treatment emergent adverse events; TK2, thymidine kinase 2; y = years; WISC = Wechsler Intelligence Scale.

Investigational Product(s)	Trial Name	Study Design	Population	Primary Outcome(s)	Status	Findings
Acipimox	AIMM: Randomised, double-blinded, placebo-controlled, adaptive design trial of the efficacy of acipimox in patients with Mitochondrial Myopathy ISRCTN12895613	Phase Ia/Ib, randomised, DB, placebo-controlled using an adaptive design [184]	≥16 y, genetically confirmed PMM. *N* = 6 acipimox; *N* = 6 placebo	ATP content in skeletal muscle	Completed	Results pending publication
AHSCT (allogeneic hematopoietic stem cell transplantation)	MASS: MNGIE Allogeneic Hematopoietic Stem Cell Transplant Safety Study NCT02427178	Phase 1	5–55 y, homozygous or compound heterozygous variants in *TYMP* *N* = 0	Engraftment success measured by neutrophil count (cells/L)	Withdrawn: poor enrolment	Results NA
Autologous mesoangioblasts (MABs)	MABS01: MABs Therapy m.3243A>G Variant Carriers NCT05063721	Open label	≥18 y, m.3243A>G variant *N* = 6 enrolled	AEs, blood flow (digital subtraction angiography) in lower leg, inflammation markers in blood and muscle	Completed	No SAEs reported. Exploratory efficacy: treated biopsies showed signals of mesoangioblast homing/migration and early muscle regeneration [185]
Bocidelpar/ASP0367 (MA-0211)	A study to evaluate ASP0367 in participants with primary mitochondrial myopathy NCT04641962	Phase 2, randomised, triple-masked, placebo-controlled	18–65 y, diagnosed with PMM *N* = 34	Distanced walked during 6MWT, no. of participants with TEAEs, no. of participants with Suicidal Ideation and/or Behaviour (C-SSRS)	Terminated: failure to meet the pre-specified criteria for efficacy	Results NA
Bezafibrate	A study of Bezafibrate in Mitochondrial Myopathy NCT02398201	Open label	18–64 y, m.3243A>G, PMM *N* = 6	Respiratory chain enzyme activity	Completed	Reduction in complex IV-deficient muscle fibres (*p* = 0.048) [186]
Coenzyme Q_10_	Phase III Trial of Coenzyme Q10 in Mitochondrial Disease NCT00432744	Phase 3, randomised, crossover, triple masking (participant, care provider, investigator)	12 m–17 y, biochemical proof of a deficiency of complex I, III or IV or molecular genetic proof of a mutation in mtDNA, or an nDNA variant in a gene known to be associated with dysfunction of the electron transport chain (e.g., *SURF1*) *N* = 24	GMFM 88; The Paediatric Quality of Life Scale	Completed	No significant difference in primary outcomes
Cysteamine bitartrate/RP103/PROCYSBI^®^	RP103-MITO-001: Open-Label, Dose-Escalating Study Assessing Safety, Tolerability, Efficacy, of RP103 in Mitochondrial Disease NCT02023866	Open label, dose-escalating	≥6 and <18 y, genetically confirmed known variant; diagnosis of inherited mitochondrial disease other than Friedreich’s ataxia *N* = 36	NPMDS score sections I–IV	Completed	Results NA
	RP103-MITO-002: A Long-term Extension of Study to Assess Cysteamine Bitartrate Delayed-release Capsules (RP103) in Children With Inherited Mitochondrial Disease NCT02473445	Phase 2, open-label extension	Completed all visits in RP103-MITO-001 (NCT02023866 *N* = 22	NPMDS score sections I–IV	Terminated: sponsor ended development of due to lack of efficacy in base study RP103-MITO-001	RP103 may reduce oxidative stress
Doxecitine and doxribtimine (MT1621/Kygevvi^®^)	RETRO: A RETROspective Study of Patients With TK2d NCT03701568	Open label (compassionate use and/or investigator-sponsored investigational new drug applications)	Treated group (*N* = 38); untreated group of patients were identified from the literature (*N* = 69)	Survival; treatment efficacy relating to motor milestones and feeding and ventilatory support; safety outcomes included treatment-emergent adverse events	Approved by FDA (2025) and EMA (2026)	Survival (death): no death in the treated group vs. 58% in the untreated group; treated patients with age of symptom onset of ≤12 years did not lose any motor milestones after treatment, and 71.4% (15/21) regained ≥1 milestone lost before treatment
	TK0102: An Open-Label Study of Continuation Treatment With Combination Pyrimidine Nucleosides in Patients With TK2 Deficiency (Continuation) NCT03845712	Phase 2, open label	Confirmed genetic variant in the *TK2* gene Child, adult, older adult *N* = 47	Safety as AEs, laboratory measurements, ECG	Active, not recruiting	Results NA
	MT-1621-104: A Study of the Efficacy and Safety of MT1621 in Thymidine Kinase 2 (TK2) Deficiency (Treatment naïve) NCT04581733	Phase 3b, open label	<18 y, diagnosis of *TK2* deficiency based on confirmed disease-causing variant(s) in the *TK2* gene *N* = 0	Proportion of subjects acquiring a motor milestone	Withdrawn: sponsor decision	Results NA
Deoxycytidine (dC) and deoxythymidine (dT)	dC-dT-MDS: Deoxynucleosides Pyrimidines as Treatment for Mitochondrial Depletion Syndrome NCT04802707	Phase 2, open label	0–60 y, diagnosis of a mitochondrial depletion disorder, pathogenic variant(s) in one of the following genes: *POLG*, *C10orf2*, *RRM2B*, *MPV17*, *SUCLA2*, *SUCLG1*, *FBXL4* Recruitment target: *N* = 200	Responder rate vs. non-responder rate—defined as having ≥2 of (1) EEG improvement, (2) decreased seizure frequency, (3) cognitive improvement, (4) caregiver impression of improvement, (5) clinical improvement, (6) normal organics and metabolism functions	Recruiting	Interim 6-month results from the first 10 participants [187]
EE-TP	TEETPIM: Trial of Erythrocyte Encapsulated Thymidine Phosphorylase In Mitochondrial Neurogastrointestinal Encephalomyopathy NCT03866954	Phase 2, open label, multiple dose	≥12 y, diagnosed with MNGIE. *N* = 0	Safety as TEAEs, laboratory measurements, vital signs, ECG, BMI, use of concomitant medication(s), pharmacodynamics	Withdrawn: change of circumstances with commercial partner	Results NA
Elamipretide/ MTP-131/ Forzinity	MMPOWER; SPIMM-201: Safety, Tolerability, and Efficacy of MTP-131 for the Treatment of Mitochondrial Myopathy NCT02367014	Phase 1/2, randomised, DB, placebo-controlled multiple ascending dose	Genetically confirmed PMM *N* = 36	Distance walked in 6MWT	Completed	Dose-dependent increase in 6MWT distance walked (*p* = 0.014); highest dose increase walk distance vs. placebo (*p* = 0.053) [188]
	MMPOWER-2; SPIMM-202: Safety, Tolerability, Efficacy of MTP-131 for Treatment of Mitochondrial Disease in Subjects From the MMPOWER Study NCT02805790	Phase 2, randomised, DB, placebo-controlled, crossover	Completed participation in the SPIMM-201 *N* = 30	Distance walked in 6MWT	Completed	Clinically meaningful increase in 6MWT did not achieve statistical significance [189]
	SPIMM-203: Open-Label Extension Trial to Characterize the Long-term Safety and Tolerability of Elamipretide in Subjects With Genetically Confirmed PMM NCT02976038	Phase 2, open-label extension study	Completed the end of study visit in SPIMM-202 *N* = 28	Distance walked in 6MWT	Terminated: registration trial did not meet the primary endpoints	Results pending publication
	MMPOWER-3; SPIMM-301: A Trial to Evaluate Safety and Efficacy of Elamipretide PMM Followed by Open-Label Extension NCT03323749	Phase 3, randomised, DB, parallel-group, placebo-controlled	≥16 and ≤80 y, genetically confirmed PMM *N* = 218	Distance walked in 6MWT; PMMSA total fatigue score	Terminated: Part1, DB portion of the trial did not meet the primary endpoints	Primary endpoints not met [190]
	NuPower; SPIMD-301: Study to Evaluate Efficacy and Safety of Elamipretide in Subjects With Primary Mitochondrial Disease From Nuclear DNA Variants (nPMD) NCT05162768	Phase 3, randomised, DB, parallel-group, placebo-controlled	≥18 and ≤70 y, nPMD, myopathy *N* = 102	Distance walked in 6MWT	Completed	Results pending publication
	TAZPOWER: A Trial to Evaluate Safety, Tolerability and Efficacy of Elamipretide in Subjects With Barth Syndrome NCT03098797	Phase 2 randomised, DB, placebo-controlled crossover	≥12 y, genetically confirmed Barth Syndrome *N* = 12	Distance walked in 6MWT; BTHS-SA total fatigue score	Completed; approved by FDA in 2025	6MWT improvements (cumulative, *p* = 0.003) BTHS-SA total fatigue scores (*p* = 0.21) [191]
	SPIES-006; SPIES-007: An Intermediate Size Expanded Access Protocol of Elamipretide NCT04689360	Expanded access	≥1 and ≤80 y or ≥12 y for Barth Syndrome in SPIES-007 Genetically confirmed PMD including Barth Syndrome	NA	Available	Results NA
	SPILH-201: A Study Investigating the Safety, Tolerability, and Efficacy of Elamipretide Topical Ophthalmic Solution for Treatment of LHON NCT02693119	Phase 2, prospective, randomised, double-masked, vehicle-controlled, plus open-label extension	≥18 and ≤50 y at the time of loss of vision in the second eye. LHON, genetic mtDNA variant m.11778G>A *N* = 12	Incidence and severity of ocular TEAEs	Completed	Well tolerated, majority of AEs were mild to moderate and resolved spontaneously [192]
Glutamine	Glutamine Supplement in MELAS Syndrome NCT04948138	Open label	≥18 y, genetically confirmed MELAS syndrome based on medical history *N* = 9	Amino acid concentration and lactate concentration in cerebrospinal fluid	Completed	Reduced CSF glutamate (*p* < 0.001) and increased CSF glutamine (*p* = 0.002); no significant change in CSF lactate [193]
	Clinical Long Term Evaluation of Glutamine Supplement in MELAS Syndrome NCT05255328	Open label	Already participated in GLN-9-MIT study	Clinical efficacy; JMDRS and MMSE	Unknown status	Results NA
Glycerol tributyrate	LHON-Plus: A Basket Clinical Study to Assess Glycerol Tributyrate in Patients With MELA) or LHON-Plus NCT06792500	Phase 1, parallel arm non-randomised dose-escalation, open label basket	18–65 y, confirmed molecular diagnosis of MELAS or LHON-Plus, symptomatic; m.3243A>G (MELAS), m.11778G>A (LHON), or a mitochondrial pathogenic variant solely mapping in a mitochondrial gene encoding a mitochondrial subunit of complex I; recruitment target: *N* = 24	Dose safety (TEAEs), potential efficacy on bioenergetic parameters for the oxidative phosphorylation pathway and/or mitochondrial ATP rate production	Recruiting	Results NA
Hydroxytyrosol	Clinical Study of Hydroxytyrosol (HT) in Mitochondrial Diseases NCT04543968	Open label	3–18 y, confirmed to have pathogenic disease-associated nDNA or mtDNA variants *N* = 9	International Paediatric Mitochondrial Disease Score (IPMDS)	Completed	Impact on PedsQL; potential effect on MELAS subgroup [194]
Idebenone/Raxone^®^	SNT-IV-003; PAROS: Post Authorisation Safety Study With Raxone in LHON Patients NCT02771379	Prospective	Prescribed Raxone^®^ for the treatment of LHON *N* = 229	Long-term safety profile assessed by incidence of AEs	Completed; approved by EMA (2015)	Results NA
	SNT-IV-005; LEROS: Study to Assess the Efficacy and Safety of Raxone in LHON Patients NCT02774005	Phase IV, open-label, natural-history-controlled	≥12 y, impaired visual acuity in affected eyes due to LHON (up to 5 years after symptom onset) *N* = 199	Proportion of eyes with clinically relevant recovery of visual acuity	Completed	Primary endpoint met, confirms long-term efficacy [126]
KL1333	KL1333 2018-102: A Phase Ia/Ib, SAD and MAD Study of KL1333 in Healthy Subjects and Patients With PMD NCT03888716	Phase 1a/b, DB, randomised, placebo-controlled, single and multiple oral dose	18–75 y, genetically confirmed PMD, clinically stable, BMI 15.0–32.0 kg/m^2^ *N* = 72 total; *N* = 8 (PMD subgroup, including *N* = 6 KL1333, *N* = 2 placebo)	Safety (incidence/severity of AEs, laboratory analytes, ECG, vital signs, physical examination)	Completed	Well tolerated, with dose-dependent gastrointestinal side effects; informed Phase 2 studies [122]
	Efficacy of KL1333 in Adult Patients With Primary Mitochondrial Disease (FALCON) NCT05650229	Phase 2, DB, randomised, placebo-controlled, flexible-dose	≥18 y, genetically confirmed PMD with multisystemic disease including: m.3243A>G, single large scale mtDNA deletion, or other multisystemic mtDNA-related disease; chronic mitochondrial fatigue; mitochondrial myopathy	PROMIS^®^ Fatigue PMD Short Form; 30sec-STS	Recruiting	Results NA
Ketogenic diet	Effectiveness of Ketogenic Diet in MELAS Syndrome NCT06013397	Open label	Diagnostic criteria of MELAS, received MELAS standard therapy but are not satisfied with the therapeutic effect, and voluntarily underwent ketogenic therapy	NMDAS, blood glucose, keto, lactate and pyruvate monitoring, HAMA, HAMD, WISC, MMSE, diagnostic evaluation (MRI, EEG)	Not yet recruiting	Results NA
L-arginine (oral and intravenous (IV)	Therapeutic regimen of L-arginine for MELAS: 9-year, prospective, multicenter, clinical research Center for Clinical Trials, Japan Medical Association MACTR-IIA00023 and JMACTR-IIA00025	OL-MELAS Research: integrated pooled data from two, 2-year, phase 3, prospective, open-label trials of oral and IV L-arginine	Oral: developed stroke-like episodes in the last 2 y, m.3243A>G, never been treated with oral L-arginine, and clinical manifestations of stroke-like episodes were evaluable IV: diagnosed with MELAS, m.3243A>G, and developed an ictus of stroke-like episodes within the previous 6 h	Oral: MELAS stroke scale IV: improvement rates of headache and nausea/vomiting at 2 h after completion of the initial IV administration	Completed	IV: improved the rates of symptoms: headache, nausea/vomiting, impaired consciousness, and visual disturbance Oral: extended the interictal phase (*p* = 0.0625) and decreased the incidence and severity of ictuses [195]
L-arginine, L-citrulline	The Effect of Arginine and Citrulline Supplementation on Endothelial Dysfunction in Mitochondrial Diseases NCT02809170	Open label, randomised crossover	3–18 y, confirmed diagnosis of mitochondrial disease with multi-organ disease involving neurological and/or muscular systems *N* = 9	Reactive hyperaemic index to reflects endothelial function, measured using the EndoPAT	Completed	Reactive hyperaemic index increased [196]
L-citrulline	L-citrulline Dose Finding Safety Study in MELAS NCT03952234	Phase 1, open label, dose-finding	18–65 y, clinical diagnosis of MELAS, m.3243A>G Recruitment target: *N* = 10	Incidence of dose-limiting toxicities to establish maximum tolerated dose	Completed	Results NA
Lenadogene nolparvovec/GS010/Lumevoq	GS-LHON/CLIN/01: Safety Evaluation of Gene Therapy in LHON Patients NCT02064569	Phase 1/2, open label, dose-escalation	≥18, genetic confirmation of m.11778G>A *ND4* LHON, visual acuity ≤ 1/10 of the less functional eye *N* = 19	Incidence of local and general AEs and SAEs	Completed	Well tolerated: 9 × 10^10^ vg/eye was identified as the best benefit/risk ratio; chosen for the subsequent studies [197]
	GS-LHON-CLIN-03A; RESCUE: Efficacy Study of GS010 for the Treatment of Vision Loss up to 6 Months From Onset in LHON Due to the ND4 Variant NCT02652767	Phase 3, randomised, DB, sham-controlled	≥15, LHON patients with vision loss ≤6 months in the first-affected eye *N* = 39	Visual acuity derived from the ETDRS chart	Completed	Primary outcome not met [174]
	GS-LHON-CLIN-03B; REVERSE: Efficacy Study of GS010 for Treatment of Vision Loss From 7 Months to 1 Year From Onset in LHON Due to the ND4 Variant NCT02652780	Phase 3, randomised, DB, sham-controlled	≥15, LHON patients with vision loss between 6 months and 1 year in both eyes *N* = 37	ETDRS Visual Acuity (Quantitative Score)	Completed	Primary endpoint was not met (*p* = 0.894) [173]
	GS-LHON-CLIN-05; REFLECT: Efficacy & Safety Study of Bilateral IVT Injection of GS010 in LHON Subjects Due to the ND4 Variant for up to 1 Year NCT03293524	Phase 3, randomised, DB, sham-controlled	≥15, clinically manifested vision loss due to m.11778G>A *ND4* LHON, to any extent, in at least one eye *N* = 98; bilateral-treated group (*N* = 48), unilateral-treated group (*N* = 50)	BCVA reported using Log of the Minimal Angle of Resolution (LogMAR)	Completed	BCVA significantly improved from baseline to 1.5 y (*p* < 0.01) [176]
	RESTORE: RESCUE and REVERSE Long-term Follow-up NCT03406104	Phase 3, prospective long-term follow-up	Treated with GS010 IVT injection in either RESCUE or REVERSE *N* = 62, including 31 from REVERSE and 31 from RESCUE	No. of eyes with ocular AEs, from 2 to 5 y post-treatment	Completed	At month 48, there was a statistically and clinically relevant difference in visual acuity of −0.33 logarithm of the minimal angle of resolution (LogMAR) (16.5 ETDRS letters equivalent) in favour of treated eyes vs. NH eyes (*p* < 0.01) [198]
	Efficacy and Safety Study of Bilateral IVT Injection of GS010 at Two Dose Levels in LHON Patients (REVISE) NCT07303296	Phase 2, open label	≥15 y, clinically manifested vision loss due to ND4 LHON in both eyes. Recruitment target: *N* = 14	BCVA change from baseline to 1.5 years post-treatment in the study eyes.	Recruiting	Results NA
Low residue diet	Low Residue Diet Study in Mitochondrial Disease NCT03388528	Phase 2, open label	≥18 y, genetic or biochemical confirmation of mitochondrial disease *N* = 24	Tolerability (via food diaries); stool consistency (Bristol Stool Form scale)	Completed	Well tolerated with no AEs; no change in stool frequency, output, or colonic transit time; proportion of stool samples with normal consistency increased (*p* = 0.01) [199]
Mavodelpar/REN001	REN001-101: A Study of the Safety of REN001 in Patients With PMM NCT03862846	Phase 1, open label	≥16 y, genetically confirmed PMM *N* = 23 enrolled	AEs as a measure of safety and tolerability	Terminated: COVID-19 sufficient data gathered to achieve the study objective	Well tolerated: all TEAEs mild-moderate; exploratory efficacy—12MWT distance increased (mean: 104 m) [200] https://doi.org/10.1038/s41598-026-43287-0
	REN001-201; STRIDE: An Efficacy and Safety Study of 24 Week Treatment With Mavodelpar (REN001) in PMM Patients NCT04535609	Randomised, DB, placebo-controlled, parallel group	≥18 y, genetically confirmed PMM *N* = 213 enrolled	Distance walked in 12MWT	Completed	Results pending publication
	Stride Ahead: REN001-202: An Open Label, Long Term Safety Study of REN001 in PMM Patients NCT05267574	Open label	Completed REN001-201 or participated in REN001-101 (mtDNA-PMM) or nDNA-PMM *N* = 155 enrolled	No. of participants with AEs and severity	Terminated: parent study failed to show therapeutic effect	Results NA
Mercaptamine-Pantetheine Disulfide Acetate/TTI-0102	Study on the Effects of TTI-0102 for Patients with MELAS Syndrome 2023-506723-28-00	Phase 2, randomised, DB, placebo-controlled	6–60 y, confirmed diagnosis of MELAS with specific genetic variants	Distance walked in 12MWT; Fatigue Severity Scale (FSS); Quality of Life questionnaire (WHOQOL-BREF) (Primary is not specified)	Not recruiting	Results NA
MNV-BM-PLC (Autologous CD34+ Cells Enriched With Placenta Derived Allogeneic Mitochondria)	PLC-PMD-01-IL: A First in Human Study to Evaluate the Safety of Infusion of MNV-BM-PLC (Autologous CD34+ Cells Enriched With Placenta Derived Allogeneic Mitochondria) in Patients With PPMD Associated With mtDNA Variant or Deletion NCT04548843	Phase I, open label, dose-escalation; sequential assignment	4–18 y, molecular diagnosis of PMD *N* = 0	No. of participants with TTEAEs assessed by CTCAE v5.0; haemoglobin; absolute neutrophil count; platelet count	Withdrawn: no recruitment	Results NA
MNV-BM-PLC	MNV-BM-BLD-001-IL: A Study to Evaluate the Safety and Therapeutic Effects of Transplantation of MNV-BM-BLD in Pediatric Patients With Pearson Syndrome NCT03384420	Phase I/2, open label, single dose	3–18 y, diagnosed with Pearson Syndrome, as verified by molecular identification of a defect in the mitochondrial DNA	No. of participants with TTEAEs; IPMDS (International Paediatric Mitochondrial Disease Scale)	Completed	Results NA
Nab-sirolimus/ABI-009	LS007. ABI-009 (Nab-sirolimus) in Patients With Genetically-confirmed Leigh or Leigh-like Syndrome NCT03747328	Phase 2a, open label	2–17 y, genetically confirmed Leigh or Leigh-like syndrome, and clinical evidence *N* = 0	Incidence of TEAEs	Withdrawn: IND withdrawn	Results NA
N-acetylcysteine (NAC)	Study of N-acetylcysteine in the Treatment of Patients With the m.3243A>G Variant and Low Brain Glutathione Levels NCT05241262	Open label	8–80 y, low brain glutathione (GSH) levels determined by MRSI, genetic confirmation of m.3243A>G Recruitment target: *N* = 18	Maximum tolerated dose of NAC	Recruiting	Results NA
Niacin/Vitamin B3	NiaMIT; NiaMIT_0001: Niacin Supplementation in Healthy Controls and Mitochondrial Myopathy Patients NCT03973203	Open label	17–80, pure mitochondrial myopathy, with no major other symptoms or manifestations, caused by single or multiple deletions of mtDNA *N* = 15	NAD+ and related metabolite levels in blood and muscle	Completed	Increased blood and muscle NAD+ of patients to the level of their controls; muscle strength and mitochondrial biogenesis increased in all subjects; muscle metabolome shifted toward controls and liver fat decreased [201]
	NiaMIT Continuation With Early-stage Mitochondrial Myopathy Patients NCT04538521	Open label	≥17, early-stage, genetically diagnosed PMM, with no major other symptoms or manifestations, caused by single or multiple deletions of mtDNA *N* = 3	NAD+ and related metabolite levels in blood and muscle	Completed	Results NA
Nicotinamide riboside/Vitamin B3	A Study to Evaluate Vitamin B3 Derivative to Treat Mitochondrial Myopathy NCT05590468	Phase 2, randomised, DB, placebo-controlled	>18 y, biochemically and/or genetically confirmed PMM Recruitment target: *N* = 34	Distance walked in 6MWT	Active, not recruiting	Results NA
	Nicotinamide Riboside and Mitochondrial Biogenesis NCT03432871	Open label	18–70 y, PEO, caused by a single deletion of mtDNA or mitochondrial disease caused by m.3243A>G or A>T variant *N* = 13	Bioavailability; safety (AEs, blood analytes, vital signs); mitochondrial biogenesis (31P-MRS, respiratory chain enzyme analysis and mtDNA quantification)	Completed	Results NA
Omaveloxolone/RTA 408/SKYCLARYS^®^	MOTOR, RTA 408-C-1403: RTA 408 Capsules in Patients With Mitochondrial Myopathy NCT02255422	Phase 2, randomised, DB, placebo-controlled, sequential	≥18 and ≤75 y, genetically confirmed PMM *N* = 53	Peak Work (Watts/kg) during exercise test	Completed	No change in primary outcome vs. placebo; highest dose reduced HR and lactate during submaximal exercise (exploratory endpoint) [202]
	A Study to Learn About the Effects and Safety of RTA 408 (Omaveloxolone) in People Aged 16 to 40 With Friedreich’s Ataxia NCT02255435	Phase 2, randomised, DB, placebo-controlled	≥16–40 y, genetically confirmed Friedreich’s ataxia *N* = 82 (FAS: 40 omaveloxolone, 42 placebo)	Peak Work (Watts/kg) during exercise test at week 12; Modified Friedreich’s Ataxia (Wek 12) Rating Scale (mFARS) at week 48	Completed; approved by FDA (2023) and EMA (2024)	Changes from baseline in mFARS scores in omaveloxolone (−1.55 ± 0.69) and placebo (0.85 ± 0.64) patients showed a difference between treatment groups of −2.40 ± 0.96 (*p* = 0.014) [203]
OMT-28	PMD-OPTION: OMT-28 in Patients With PMD NCT05972954	Phase 2a, open label	18–60 y, variant resulting in PMD, including m3243A>G, m8344A>G, and single mtDNA deletions; diagnosis of cardiomyopathy as defined, myopathy *N* = 28	No. of TEAEs; no. patients with a between-phase difference in GDF-15 of ≥20% decrease	Completed	Results NA
rAAV2-*ND4*	A Single Intravitreal Injection of rAAV2-ND4 for the Treatment of LHON NCT03153293	Phase 2/3, open label	10–65 y, LHON and m.11778G>A *N* = 149	BCVA, computerised visual field	Unknown	Rapid and significant BCVA improvement reported within 3 days in at least 1 eye of 36.2% of patients and in both eyes in 11.4% of patients [204]
Resveratrol	Resveratrol Supplementation in Patients With Mitochondrial Myopathies and Skeletal Muscle Fatty Acid Oxidation Disorders NCT03728777	DB, placebo-controlled, crossover	≥18 and ≤80 y, genetically verified mitochondrial disorder or a fatty acid oxidation deficiency *N* = 11	Decrease in HR during constant load cycling exercise	Completed	No significant differences between treatments [205].
RG2133 (2′,3′,5′-tri-O-acetyluridine)	RG2133 (2′,3′,5′-Tri-O-Acetyluridine) in Mitochondrial Disease NCT00060515	Phase 1, dose-escalation	≥3 y, mitochondrial disease *N* = 12	NA	Terminated	Results NA
scAAV2-P1ND4v2 (AAV-*ND4*)	Safety Study of an Adeno-associated Virus Vector for Gene Therapy of LHON NCT02161380	Phase 1, open label, dose-escalation	≥15 y, LHON and m.11778G>A	No. of treatment-related AEs	Completed	No SAEs, mild transient side effects observed [206]
SPP-004 (5-aminolevulinic acid hydrochloride + sodium ferrous citrate)	jRCT2091220200 (SPED-ALA-001)	Randomised, DB, placebo-controlled	2–3 y, confirmed respiratory chain enzyme deficiency or mitochondrial gene abnormality *N* = 10 (*N* = 5 SPP-004, *N* = 5 placebo)	NPMDS Section I–III	Completed	No significant differences between groups [207]
	jRCT2091220214 (SPED-ALA-002	Open label	Completed SPED-ALA-001 *N* = 10	NPMDS Section I–III	Completed	Stabilised or improved scores [207]
Sonlicromanol/ KH176	KH-176-202: The KHENERGYZE Study NCT04165239	Phase 2b, DB, open-label, randomised, placebo-controlled, three-way crossover	≥18 y, confirmed m.3243A>G variant and clinical signs of mitochondrial disease *N* = 24	Attention domain score of cognitive functioning, (visual Identification Test of the Cogstate computerised cognitive testing battery)	Completed	Primary outcome did not reach statistical significance [208]
	KH176-203; The KHENEREXT Study NCT04604548	Phase 2, open label	Completed KH176-202 *N* = 12	Frequency of TEAEs	Completed	Good tolerability with favourable safety [209]
	KH176-204; The KHENERGYC Study NCT04846036	Phase 2, randomised, DB, placebo controlled, parallel-group	0 m–17 y, genetically confirmed PMD Recruitment target: *N* = 24	Motor Symptom Severity as assessed with the GMFM-88	Suspended: strategic company decision—not related to safety concerns	Results NA
	KH176-301; KHENERFIN: A Trial to Evaluate the Efficacy and Safety of Sonlicromanol in PMD NCT06451757	Phase 3, randomised, DB, placebo-controlled, parallel-group	≥18 y, confirmed m.3243A>G Recruitment target: *N* = 220	Neuro-QoL Fatigue SF v1; time to complete 5XSST	Recruiting	Results NA
Thymidine	AAAQ7552: Treatment of TK2 Deficiency with Thymidine and Deoxycytidine NCT03639701	Phase 1/2½, open label	Genetically confirmed diagnosis of *TK2* deficiency, symptomatic. *N* = 23	Blood analysis (ALT, AST, GGT, lymphocyte count, creatinine), ECG, PROMIS scale v1.0—Gastrointestinal Diarrhea 6a score	Active, not recruiting	Results NA
Transcranial direct current stimulation (tDCS)	TRANscranial direct current Stimulation for FOcal Refractory epilepsy in Mitochondrial disease (TRANSFORM): delayed-start, randomised, double-blinded, placebo-controlled trial	Delayed-start, randomised, double blind, sham-controlled design [210]	Confirmed diagnosis of mitochondrial disease with drug-resistant focal epilepsy aged ≥ 2 years	>50% reduction from baseline in seizure frequency	Recruiting	Results NA
Vatiquinone/EPI-743	EPI743-12-002: Safety and Efficacy Study of EPI-743 in Children With Leigh Syndrome NCT01721733	Phase 2B, randomised, DB, placebo controlled with an extension phase	<18 y, clinical and MRI diagnosis of Leigh syndrome	Change in NPMDS score from baseline to 6 months comparing to placebo	Completed	Results NA
	EPI-2009-1: EPI-743 for Mitochondrial Respiratory Chain Diseases NCT01370447	Emergency use	Genetically confirmed mitochondrial respiratory chain disease, deemed to be within 90 days of end-of-life care		No longer available	Results NA
	EPI743-12-002: Safety and Efficacy Study of EPI-743 in Children With Leigh Syndrome NCT01721733	Phase 2B, randomised, DB, placebo-controlled	<18 y, clinical and MRI diagnosis of Leigh syndrome		Completed	Fewer patients requiring hospitalisation or experiencing SAEs
	EPI743-13-023 Long-Term Safety and Efficacy Evaluation of EPI-743 in Children with Leigh Syndrome NCT02352896	Open label	Completed EPI743-12-002 *N* = 30	NPMDS sections 1–3; dose-limiting SAEs	Completed	Progressive decline in hospitalisations and SAEs
	Phase 2 Study of EPI-743 in Children With Pearson Syndrome NCT02104336	Phase 2, open label	<18 y, genetically confirmed Pearson syndrome	Occurrence of episodes of sepsis, metabolic crisis or hepatic failure	Terminated: results from other studies did not support continuation	Results NA
	PTC743-MIT-001-EP: A Study to Evaluate Efficacy and Safety of Vatiquinone for Treating Mitochondrial Disease in Participants With Refractory Epilepsy (MIT-E) NCT04378075	Phase 2/3, parallel-arm, DB, placebo-controlled	Genetic confirmation of inherited mitochondrial disease with associated epilepsy phenotype *N* = 68	No. of observable motor seizures per 28 days	Terminated: sponsor decision	Results NA
	A Safety Study for Previously Treated Vatiquinone (PTC743) Participants With Inherited Mitochondrial Disease NCT05218655	Phase 3, open label	Completed previous vatiquinone clinical study or treatment plan *N* = 101	No. of participants with TEAEs	Completed	Results NA
Zagociguat/ IW-6463/CY6463	Phase 2a Study of IW-6463 in Adults Diagnosed With MELAS NCT04475549	Phase 2a, open label	≥18 y, genetic confirmation of a known mitochondrial disease variant, neurological features of MELAS *N* = 8	No. of participants with study drug dose reductions or discontinuations due to ≥1 TEAE; no. of participants with ≥1 AE, TEAE, SAE or AESI	Terminated due to enrolment challenges	Results NA
	PRIZM A Phase 2b Study of Zagociguat in Patients with MELAS NCT06402123	Phase 2b, randomised, DB, placebo-controlled crossover	18–75 y, diagnosed with MELAS based on the presence of each of: documented pathogenic variant in a mtDNA gene and, history of ≥ 1 stroke-like episodes with MRI findings consistent with stroke-like lesions	TEAEs incidence; PROMIS Fatigue MELAS SF scores; Groton Maze Learning scores; International digit symbol substitution test scores	Recruiting	Results NA

## Data Availability

No new data were created or analysed in this study.

## References

[1] Ng Y.S., McFarland R. (2023). Mitochondrial encephalomyopathy. Handbook of Clinical Neurology.

[2] Ng Y.S., Bindoff L.A., Gorman G.S., Klopstock T., Kornblum C., Mancuso M., McFarland R., Sue C.M., Suomalainen A., Taylor R.W. (2021). Mitochondrial disease in adults: Recent advances and future promise. Lancet. Neurol..

[3] Gorman G.S., Chinnery P.F., DiMauro S., Hirano M., Koga Y., McFarland R., Suomalainen A., Thorburn D.R., Zeviani M., Turnbull D.M. (2016). Mitochondrial diseases. Nat. Rev. Dis. Primers.

[4] Mancuso M., Papadopoulou M.T., Ng Y.S., Ardissone A., Bellusci M., Bertini E., Di Vito L., Evangelista T., Fons C., Hikmat O. (2024). Management of seizures in patients with primary mitochondrial diseases: Consensus statement from the InterERNs Mitochondrial Working Group. Eur. J. Neurol..

[5] Mancuso M., Lopriore P., Semmler L., Kornblum C. (2025). 280th ENMC International Workshop: The ERN EURO-NMD mitochondrial diseases working group; diagnostic criteria and outcome measures in primary mitochondrial myopathies. Hoofddorp, The Netherlands, 22–24 November 2024. Neuromuscul. Disord. NMD.

[6] Diodato D., Schiff M., Cohen B.H., Bertini E., Rahman S. (2023). 258th ENMC international workshop Leigh syndrome spectrum: Genetic causes, natural history and preparing for clinical trials 25–27 March 2022, Hoofddorp, Amsterdam, The Netherlands. Neuromuscul. Disord. NMD.

[7] Pitceathly R.D.S., Keshavan N., Rahman J., Rahman S. (2021). Moving towards clinical trials for mitochondrial diseases. J. Inherit. Metab. Dis..

[8] Rath S., Sharma R., Gupta R., Ast T., Chan C., Durham T.J., Goodman R.P., Grabarek Z., Haas M.E., Hung W.H.W. (2021). MitoCarta3.0: An updated mitochondrial proteome now with sub-organelle localization and pathway annotations. Nucleic Acids Res..

[9] Ferreira C.R., Rahman S., Keller M., Zschocke J. (2021). An international classification of inherited metabolic disorders (ICIMD). J. Inherit. Metab. Dis..

[10] Messina M., Ganetzky R., Ferreira C.R., Blau N., Rahman S. (2026). Clinical and biochemical footprints of primary mitochondrial disorders: Proposed nosology. Mol. Genet. Metab..

[11] Alston C.L., Stenton S.L., Hudson G., Prokisch H., Taylor R.W. (2021). The genetics of mitochondrial disease: Dissecting mitochondrial pathology using multi-omic pipelines. J. Pathol..

[12] Gorman G.S., Schaefer A.M., Ng Y., Gomez N., Blakely E.L., Alston C.L., Feeney C., Horvath R., Yu-Wai-Man P., Chinnery P.F. (2015). Prevalence of nuclear and mitochondrial DNA mutations related to adult mitochondrial disease. Ann. Neurol..

[13] Stenton S.L., Prokisch H. (2020). Genetics of mitochondrial diseases: Identifying mutations to help diagnosis. EBioMedicine.

[14] Schon K.R., Horvath R., Wei W., Calabrese C., Tucci A., Ibañez K., Ratnaike T., Pitceathly R.D.S., Bugiardini E., Quinlivan R. (2021). Use of whole genome sequencing to determine genetic basis of suspected mitochondrial disorders: Cohort study. BMJ (Clin. Res. Ed.).

[15] Davis R.L., Kumar K.R., Puttick C., Liang C., Ahmad K.E., Edema-Hildebrand F., Park J.S., Minoche A.E., Gayevskiy V., Mallawaarachchi A.C. (2022). Use of Whole-Genome Sequencing for Mitochondrial Disease Diagnosis. Neurology.

[16] Rius R., Compton A.G., Baker N.L., Balasubramaniam S., Best S., Bhattacharya K., Boggs K., Boughtwood T., Braithwaite J., Bratkovic D. (2025). The Australian Genomics Mitochondrial Flagship: A national program delivering mitochondrial diagnoses. Genet. Med. Off. J. Am. Coll. Med. Genet..

[17] Ball M., Baker N., Lim S.C., Casauria S., Lunke S., Compton A.G., Thorburn D.R., Christodoulou J., Stark Z. (2026). Mainstreaming genomic testing for mitochondrial disease in Australia. Eur. J. Hum. Genet..

[18] Wortmann S.B., Koolen D.A., Smeitink J.A., van den Heuvel L., Rodenburg R.J. (2015). Whole exome sequencing of suspected mitochondrial patients in clinical practice. J. Inherit. Metab. Dis..

[19] Pronicka E., Piekutowska-Abramczuk D., Ciara E., Trubicka J., Rokicki D., Karkucińska-Więckowska A., Pajdowska M., Jurkiewicz E., Halat P., Kosińska J. (2016). New perspective in diagnostics of mitochondrial disorders: Two years’ experience with whole-exome sequencing at a national paediatric centre. J. Transl. Med..

[20] Kohda M., Tokuzawa Y., Kishita Y., Nyuzuki H., Moriyama Y., Mizuno Y., Hirata T., Yatsuka Y., Yamashita-Sugahara Y., Nakachi Y. (2016). A Comprehensive Genomic Analysis Reveals the Genetic Landscape of Mitochondrial Respiratory Chain Complex Deficiencies. PLoS Genet..

[21] Legati A., Reyes A., Nasca A., Invernizzi F., Lamantea E., Tiranti V., Garavaglia B., Lamperti C., Ardissone A., Moroni I. (2016). New genes and pathomechanisms in mitochondrial disorders unraveled by NGS technologies. Biochim. Biophys. Acta.

[22] Puusepp S., Reinson K., Pajusalu S., Murumets Ü., Õiglane-Shlik E., Rein R., Talvik I., Rodenburg R.J., Õunap K. (2018). Effectiveness of whole exome sequencing in unsolved patients with a clinical suspicion of a mitochondrial disorder in Estonia. Mol. Genet. Metab. Rep..

[23] Theunissen T.E.J., Nguyen M., Kamps R., Hendrickx A.T., Sallevelt S., Gottschalk R.W.H., Calis C.M., Stassen A.P.M., de Koning B., Mulder-Den Hartog E.N.M. (2018). Whole Exome Sequencing Is the Preferred Strategy to Identify the Genetic Defect in Patients with a Probable or Possible Mitochondrial Cause. Front. Genet..

[24] Grigalionienė K., Burnytė B., Ambrozaitytė L., Utkus A. (2023). Wide diagnostic and genotypic spectrum in patients with suspected mitochondrial disease. Orphanet J. Rare Dis..

[25] Riley L.G., Cowley M.J., Gayevskiy V., Minoche A.E., Puttick C., Thorburn D.R., Rius R., Compton A.G., Menezes M.J., Bhattacharya K. (2020). The diagnostic utility of genome sequencing in a pediatric cohort with suspected mitochondrial disease. Genet. Med..

[26] Tsang M.H.Y., Kwong A.K.Y., Chan K.L.S., Fung J.L.F., Yu M.H.C., Mak C.C.Y., Yeung K.S., Rodenburg R.J.T., Smeitink J.A.M., Chan R. (2020). Delineation of molecular findings by whole-exome sequencing for suspected cases of paediatric-onset mitochondrial diseases in the Southern Chinese population. Hum. Genom..

[27] Forny P., Footitt E., Davison J.E., Lam A., Woodward C.E., Batzios S., Bhate S., Chakrapani A., Cleary M., Gissen P. (2021). Diagnosing Mitochondrial Disorders Remains Challenging in the Omics Era. Neurol. Genet..

[28] Yépez V.A., Gusic M., Kopajtich R., Mertes C., Smith N.H., Alston C.L., Ban R., Beblo S., Berutti R., Blessing H. (2022). Clinical implementation of RNA sequencing for Mendelian disease diagnostics. Genome Med..

[29] Wu T.H., Peng J., Yang L., Chen Y.H., Lu X.L., Huang J.T., You J.Y., Ou-Yang W.X., Sun Y.Y., Xue Y.N. (2023). Use of dual genomic sequencing to screen mitochondrial diseases in pediatrics: A retrospective analysis. Sci. Rep..

[30] Rouzier C., Pion E., Chaussenot A., Bris C., Ait-El-Mkadem Saadi S., Desquiret-Dumas V., Gueguen N., Fragaki K., Amati-Bonneau P., Barcia G. (2024). Primary mitochondrial disorders and mimics: Insights from a large French cohort. Ann. Clin. Transl. Neurol..

[31] Ouyang X., Chi D., Zhang Y., Yu T., Zhang Q., Xu L., Zhang V.W., Wang B. (2025). Application of rapid clinical exome sequencing technology in the diagnosis of critically ill pediatric patients with suspected genetic diseases. Front. Genet..

[32] Ambrose A., Bahl S., Sharma S., Zhang D., Hung C., Jain-Ghai S., Chan A., Mercimek-Andrews S. (2024). Genetic landscape of primary mitochondrial diseases in children and adults using molecular genetics and genomic investigations of mitochondrial and nuclear genome. Orphanet J. Rare Dis..

[33] Hock D.H., Caruana N.J., Semcesen L.N., Lake N.J., Formosa L.E., Amarasekera S.S.C., Stait T., Tregoning S., Frajman L.E., Bournazos A.M. (2025). Untargeted proteomics enables ultra-rapid variant prioritisation in mitochondrial and other rare diseases. Genome Med..

[34] Liu Z., Duan X., Peymani F., Wang J., Bao C., Xu C., Zou Y., Zhang Z., Zhang Y., Li T. (2026). RNA Sequencing Resolves Cryptic Pathogenic Variants in Mitochondrial Disease. Ann. Clin. Transl. Neurol..

[35] Kansal R. (2025). Rapid Whole-Genome Sequencing in Critically Ill Infants and Children with Suspected, Undiagnosed Genetic Diseases: Evolution to a First-Tier Clinical Laboratory Test in the Era of Precision Medicine. Children.

[36] Lunke S., Bouffler S.E., Patel C.V., Sandaradura S.A., Wilson M., Pinner J., Hunter M.F., Barnett C.P., Wallis M., Kamien B. (2023). Integrated multi-omics for rapid rare disease diagnosis on a national scale. Nat. Med..

[37] Maron J.L., Kingsmore S., Gelb B.D., Vockley J., Wigby K., Bragg J., Stroustrup A., Poindexter B., Suhrie K., Kim J.H. (2023). Rapid Whole-Genomic Sequencing and a Targeted Neonatal Gene Panel in Infants with a Suspected Genetic Disorder. Jama.

[38] Olimpio C., Paramonov I., Matalonga L., Laurie S., Schon K., Polavarapu K., Kirschner J., Schara-Schmidt U., Lochmüller H., Chinnery P.F. (2024). Increased Diagnostic Yield by Reanalysis of Whole Exome Sequencing Data in Mitochondrial Disease. J. Neuromuscul. Dis..

[39] Ratnaike T., Paramonov I., Olimpio C., Hoischen A., Beltran S., Matalonga L., Horváth R. (2025). Mitochondrial DNA disease discovery through evaluation of genotype and phenotype data: The Solve-RD experience. Am. J. Hum. Genet..

[40] Stenton S.L., Laricchia K., Lake N.J., Chaluvadi S., Ganesh V., DiTroia S., Osei-Owusu I., Pais L., O’Heir E., Austin-Tse C. (2025). Mitochondrial DNA variant detection in over 6,500 rare disease families by the systematic analysis of exome and genome sequencing data resolves undiagnosed cases. HGG Adv..

[41] Bagger F.O., Borgwardt L., Jespersen A.S., Hansen A.R., Bertelsen B., Kodama M., Nielsen F.C. (2024). Whole genome sequencing in clinical practice. BMC Med. Genom..

[42] Wei W., Pagnamenta A.T., Gleadall N., Sanchis-Juan A., Stephens J., Broxholme J., Tuna S., Odhams C.A., Ambrose J.C., Baple E.L. (2020). Nuclear-mitochondrial DNA segments resemble paternally inherited mitochondrial DNA in humans. Nat. Commun..

[43] Logsdon G.A., Vollger M.R., Eichler E.E. (2020). Long-read human genome sequencing and its applications. Nat. Rev. Genet..

[44] Höps W., Weiss M.M., Derks R., Galbany J.C., Ouden A.D., van den Heuvel S., Timmermans R., Smits J., Mokveld T., Dolzhenko E. (2025). HiFi long-read genomes for difficult-to-detect, clinically relevant variants. Am. J. Hum. Genet..

[45] Steyaert W., Sagath L., Demidov G., Yépez V.A., Esteve-Codina A., Gagneur J., Ellwanger K., Derks R., Weiss M., den Ouden A. (2025). Unraveling undiagnosed rare disease cases by HiFi long-read genome sequencing. Genome Res..

[46] Burke W., Parens E., Chung W.K., Berger S.M., Appelbaum P.S. (2022). The Challenge of Genetic Variants of Uncertain Clinical Significance: A Narrative Review. Ann. Intern. Med..

[47] Schon K.R., Ratnaike T., van den Ameele J., Horvath R., Chinnery P.F. (2020). Mitochondrial Diseases: A Diagnostic Revolution. Trends Genet..

[48] Wortmann S.B., Mayr J.A., Nuoffer J.M., Prokisch H., Sperl W. (2017). A Guideline for the Diagnosis of Pediatric Mitochondrial Disease: The Value of Muscle and Skin Biopsies in the Genetics Era. Neuropediatrics.

[49] Mavraki E., Labrum R., Sergeant K., Alston C.L., Woodward C., Smith C., Knowles C.V.Y., Patel Y., Hodsdon P., Baines J.P. (2023). Genetic testing for mitochondrial disease: The United Kingdom best practice guidelines. Eur. J. Hum. Genet..

[50] Kremer L.S., Bader D.M., Mertes C., Kopajtich R., Pichler G., Iuso A., Haack T.B., Graf E., Schwarzmayr T., Terrile C. (2017). Genetic diagnosis of Mendelian disorders via RNA sequencing. Nat. Commun..

[51] Correia S.P., Moedas M.F., Taylor L.S., Naess K., Lim A.Z., McFarland R., Kazior Z., Rumyantseva A., Wibom R., Engvall M. (2024). Quantitative proteomics of patient fibroblasts reveal biomarkers and diagnostic signatures of mitochondrial disease. JCI Insight.

[52] Jobanputra V., Schroeder B., Rehm H.L., Shen W., Spiteri E., Nakouzi G., Taylor S., Marshall C.R., Meng L., Kingsmore S.F. (2024). Advancing access to genome sequencing for rare genetic disorders: Recent progress and call to action. NPJ Genom. Med..

[53] Munung N.S. (2025). Science and Society: Pathways to Equitable Access and Delivery of Genomics Medicine in Africa. Curr. Genet. Med. Rep..

[54] Tekola-Ayele F., Rotimi C.N. (2015). Translational Genomics in Low- and Middle-Income Countries: Opportunities and Challenges. Public Health Genom..

[55] Poli M.C., Rebolledo-Jaramillo B., Lagos C., Orellana J., Moreno G., Martín L.M., Encina G., Böhme D., Faundes V., Zavala M.J. (2024). Decoding complex inherited phenotypes in rare disorders: The DECIPHERD initiative for rare undiagnosed diseases in Chile. Eur. J. Hum. Genet..

[56] Hubens W.H.G., Vallbona-Garcia A., de Coo I.F.M., van Tienen F.H.J., Webers C.A.B., Smeets H.J.M., Gorgels T. (2022). Blood biomarkers for assessment of mitochondrial dysfunction: An expert review. Mitochondrion.

[57] Thompson J.L.P., Karaa A., Pham H., Yeske P., Krischer J., Xiao Y., Long Y., Kramer A., Dimmock D., Holbert A. (2023). The evolution of the mitochondrial disease diagnostic odyssey. Orphanet J. Rare Dis..

[58] Kharitonenkov A., Shiyanova T.L., Koester A., Ford A.M., Micanovic R., Galbreath E.J., Sandusky G.E., Hammond L.J., Moyers J.S., Owens R.A. (2005). FGF-21 as a novel metabolic regulator. J. Clin. Investig..

[59] Suomalainen A., Elo J.M., Pietiläinen K.H., Hakonen A.H., Sevastianova K., Korpela M., Isohanni P., Marjavaara S.K., Tyni T., Kiuru-Enari S. (2011). FGF-21 as a biomarker for muscle-manifesting mitochondrial respiratory chain deficiencies: A diagnostic study. Lancet Neurol..

[60] Yatsuga S., Fujita Y., Ishii A., Fukumoto Y., Arahata H., Kakuma T., Kojima T., Ito M., Tanaka M., Saiki R. (2015). Growth differentiation factor 15 as a useful biomarker for mitochondrial disorders. Ann. Neurol..

[61] Lin Y., Ji K., Ma X., Liu S., Li W., Zhao Y., Yan C. (2020). Accuracy of FGF-21 and GDF-15 for the diagnosis of mitochondrial disorders: A meta-analysis. Ann. Clin. Transl. Neurol..

[62] Fernández A.C., Estrella J., Oglesbee D., Larson A.A., Van Hove J.L.K. (2025). The clinical utility in hospital-wide use of growth differentiation factor 15 as a biomarker for mitochondrial DNA-related disorders. J. Inherit. Metab. Dis..

[63] Bermejo-Guerrero L., de Fuenmayor-Fernández de la Hoz C.P., Guerrero-Molina M.P., Martín-Jiménez P., Blázquez A., Serrano-Lorenzo P., Lora D., Morales-Conejo M., González-Martínez I., López-Jiménez E.A. (2023). Serum GDF-15 Levels Accurately Differentiate Patients with Primary Mitochondrial Myopathy, Manifesting with Exercise Intolerance and Fatigue, from Patients with Chronic Fatigue Syndrome. J. Clin. Med..

[64] Davis R.L., Liang C., Sue C.M. (2016). A comparison of current serum biomarkers as diagnostic indicators of mitochondrial diseases. Neurology.

[65] Lehtonen J.M., Auranen M., Darin N., Sofou K., Bindoff L., Hikmat O., Uusimaa J., Vieira P., Tulinius M., Lönnqvist T. (2021). Diagnostic value of serum biomarkers FGF21 and GDF15 compared to muscle sample in mitochondrial disease. J. Inherit. Metab. Dis..

[66] Tsygankova P.G., Itkis Y.S., Krylova T.D., Kurkina M.V., Bychkov I.O., Ilyushkina A.A., Zabnenkova V.V., Mikhaylova S.V., Pechatnikova N.L., Sheremet N.L. (2019). Plasma FGF-21 and GDF-15 are elevated in different inherited metabolic diseases and are not diagnostic for mitochondrial disorders. J. Inherit. Metab. Dis..

[67] Lehtonen J.M., Forsström S., Bottani E., Viscomi C., Baris O.R., Isoniemi H., Höckerstedt K., Österlund P., Hurme M., Jylhävä J. (2016). FGF21 is a biomarker for mitochondrial translation and mtDNA maintenance disorders. Neurology.

[68] Li Y., Li S., Qiu Y., Zhou M., Chen M., Hu Y., Hong S., Jiang L., Guo Y. (2022). Circulating FGF21 and GDF15 as Biomarkers for Screening, Diagnosis, and Severity Assessment of Primary Mitochondrial Disorders in Children. Front. Pediatr..

[69] Davis R.L., Liang C., Edema-Hildebrand F., Riley C., Needham M., Sue C.M. (2013). Fibroblast growth factor 21 is a sensitive biomarker of mitochondrial disease. Neurology.

[70] Koene S., de Laat P., van Tienoven D.H., Vriens D., Brandt A.M., Sweep F.C., Rodenburg R.J., Donders A.R., Janssen M.C., Smeitink J.A. (2014). Serum FGF21 levels in adult m. 3243A>G carriers: Clinical implications. Neurology.

[71] Montano V., Gruosso F., Carelli V., Comi G.P., Filosto M., Lamperti C., Mongini T., Musumeci O., Servidei S., Tonin P. (2020). Primary mitochondrial myopathy: Clinical features and outcome measures in 118 cases from Italy. Neurol. Genet..

[72] Koene S., de Laat P., van Tienoven D.H., Weijers G., Vriens D., Sweep F.C., Timmermans J., Kapusta L., Janssen M.C., Smeitink J.A. (2015). Serum GDF15 Levels Correlate to Mitochondrial Disease Severity and Myocardial Strain, but Not to Disease Progression in Adult m. 3243A>G Carriers. JIMD Rep..

[73] Peretz R.H., Ah Mew N., Vernon H.J., Ganetzky R.D. (2021). Prospective diagnosis of MT-ATP6-related mitochondrial disease by newborn screening. Mol. Genet. Metab..

[74] Tise C.G., Verscaj C.P., Mendelsohn B.A., Woods J., Lee C.U., Enns G.M., Stander Z., Hall P.L., Cowan T.M., Cusmano-Ozog K.P. (2023). MT-ATP6 mitochondrial disease identified by newborn screening reveals a distinct biochemical phenotype. Am. J. Med. Genet. A.

[75] Martín-Jimenez P., Bermejo-Guerrero L., Navarro-Riquelme M., Serrano-Lorenzo P., Garrido-Moraga R., Hernández-Laín A., Hernández-Voth A., Lora D., Morales M., Arenas J. (2025). Comprehensive analysis of GDF15 as a biomarker in primary mitochondrial myopathies. Mol. Genet. Metab..

[76] Dominguez-Gonzalez C., Badosa C., Madruga-Garrido M., Martí I., Paradas C., Ortez C., Diaz-Manera J., Berardo A., Alonso-Pérez J., Trifunov S. (2020). Growth Differentiation Factor 15 is a potential biomarker of therapeutic response for TK2 deficient myopathy. Sci. Rep..

[77] Huang Q., Trumpff C., Monzel A.S., Rausser S., Devine J., Liu C.C., Kelly C., Kurade M., Li S., Engelstad K. (2025). The mitochondrial disease biomarker GDF15 is dynamic, quantifiable in saliva, and correlates with disease severity. Mol. Genet. Metab..

[78] Alagaratnam J., von Widekind S., De Francesco D., Underwood J., Edison P., Winston A., Zetterberg H., Fidler S. (2021). Correlation between CSF and blood neurofilament light chain protein: A systematic review and meta-analysis. BMJ Neurol. Open.

[79] Gaetani L., Blennow K., Calabresi P., Di Filippo M., Parnetti L., Zetterberg H. (2019). Neurofilament light chain as a biomarker in neurological disorders. J. Neurol. Neurosurg. Psychiatry.

[80] Varhaug K.N., Hikmat O., Nakkestad H.L., Vedeler C.A., Bindoff L.A. (2021). Serum biomarkers in primary mitochondrial disorders. Brain Commun..

[81] Zheng Y.S., Sun C., Wang R., Chen N., Luo S.S., Xi J.Y., Lu J.H., Zhao C.B., Li Y.X., Zhou L. (2021). Neurofilament light is a novel biomarker for mitochondrial encephalomyopathy, lactic acidosis, and stroke-like episodes. Sci. Rep..

[82] Maresca A., Moresco M., Amore G., La Morgia C., Valentino M.L., Capirossi G., Sacchetti G., Carelli V., Vedeler C., Bindoff L.A. (2026). Biomarking MELAS with neurofilament light chain and circulating cell free mitochondrial DNA. Mol. Genet. Metab..

[83] Bermejo-Guerrero L., Restrepo-Vera J.L., Martin-Jimenez P., Navarro-Riquelme M., Garrido-Moraga R., Ochoa L.E., González-Quintana A., Hernandez-Lain A., Castillo-Villalba J., Morís G. (2026). Clinical Heterogeneity and Candidate Biomarkers in POLG-Related Mitochondrial Disease. Neurol. Genet..

[84] Rabier D., Diry C., Rotig A., Rustin P., Heron B., Bardet J., Parvy P., Ponsot G., Marsac C., Saudubray J.M. (1998). Persistent hypocitrullinaemia as a marker for mtDNA NARP T 8993 G mutation?. J. Inherit. Metab. Dis..

[85] Carli S., Levarlet A., Diodato D., Bertini E.S., Martinelli D., Malandrini A., Lopergolo D., Gallus G.N., Ganetzky R.D., La Morgia C. (2025). Natural History of Patients with Mitochondrial ATPase Deficiency Due to Pathogenic Variants of MT-ATP6 and MT-ATP8. Neurology.

[86] Zhang A., Sun H., Yan G., Wang P., Wang X. (2016). Mass spectrometry-based metabolomics: Applications to biomarker and metabolic pathway research. Biomed. Chromatogr..

[87] Rahman J., Rahman S. (2018). Mitochondrial medicine in the omics era. Lancet.

[88] Gervasoni J., Primiano A., Cicchinelli M., Santucci L., Servidei S., Urbani A., Primiano G., Iavarone F. (2024). Mitochondrial Biomarkers in the Omics Era: A Clinical-Pathophysiological Perspective. Int. J. Mol. Sci..

[89] Sharma R., Reinstadler B., Engelstad K., Skinner O.S., Stackowitz E., Haller R.G., Clish C.B., Pierce K., Walker M.A., Fryer R. (2021). Circulating markers of NADH-reductive stress correlate with mitochondrial disease severity. J. Clin. Investig..

[90] Buzkova J., Nikkanen J., Ahola S., Hakonen A.H., Sevastianova K., Hovinen T., Yki-Järvinen H., Pietiläinen K.H., Lönnqvist T., Velagapudi V. (2018). Metabolomes of mitochondrial diseases and inclusion body myositis patients: Treatment targets and biomarkers. EMBO Mol. Med..

[91] de Laat P., Koene S., van den Heuvel L.P., Rodenburg R.J., Janssen M.C., Smeitink J.A. (2012). Clinical features and heteroplasmy in blood, urine and saliva in 34 Dutch families carrying the m. 3243A > G mutation. J. Inherit. Metab. Dis..

[92] Nesbitt V., Pitceathly R.D.S., Turnbull D.M., Taylor R.W., Sweeney M.G., Mudanohwo E.E., Rahman S., Hanna M.G., McFarland R. (2013). The UK MRC Mitochondrial Disease Patient Cohort Study: Clinical phenotypes associated with the m. 3243A>G mutation—Implications for diagnosis and management. J. Neurol. Neurosurg. Psychiatry.

[93] Ng Y.S., Lax N.Z., Blain A.P., Erskine D., Baker M.R., Polvikoski T., Thomas R.H., Morris C.M., Lai M., Whittaker R.G. (2022). Forecasting stroke-like episodes and outcomes in mitochondrial disease. Brain.

[94] Grady J.P., Pickett S.J., Ng Y.S., Alston C.L., Blakely E.L., Hardy S.A., Feeney C.L., Bright A.A., Schaefer A.M., Gorman G.S. (2018). mtDNA heteroplasmy level and copy number indicate disease burden in m. 3243A > G mitochondrial disease. EMBO Mol. Med..

[95] Grady J.P., Campbell G., Ratnaike T., Blakely E.L., Falkous G., Nesbitt V., Schaefer A.M., McNally R.J., Gorman G.S., Taylor R.W. (2014). Disease progression in patients with single, large-scale mitochondrial DNA deletions. Brain.

[96] Bernardino Gomes T.M., Ng Y.S., Pickett S.J., Turnbull D.M., Vincent A.E. (2021). Mitochondrial DNA disorders: From pathogenic variants to preventing transmission. Hum. Mol. Genet..

[97] Pickett S.J., Grady J.P., Ng Y.S., Gorman G.S., Schaefer A.M., Wilson I.J., Cordell H.J., Turnbull D.M., Taylor R.W., McFarland R. (2018). Phenotypic heterogeneity in m. 3243A>G mitochondrial disease: The role of nuclear factors. Ann. Clin. Transl. Neurol..

[98] Carelli V., d’Adamo P., Valentino M.L., La Morgia C., Ross-Cisneros F.N., Caporali L., Maresca A., Loguercio Polosa P., Barboni P., De Negri A. (2016). Parsing the differences in affected with LHON: Genetic versus environmental triggers of disease conversion. Brain.

[99] Blickhäuser B., Stenton S.L., Neuhofer C.M., Floride E., Nesbitt V., Fratter C., Koch J., Kauffmann B., Catarino C., Schlieben L.D. (2024). Digenic Leigh syndrome on the background of the m. 11778G>A Leber hereditary optic neuropathy variant. Brain.

[100] Whittaker R.G., Devine H.E., Gorman G.S., Schaefer A.M., Horvath R., Ng Y., Nesbitt V., Lax N.Z., McFarland R., Cunningham M.O. (2015). Epilepsy in adults with mitochondrial disease: A cohort study. Ann. Neurol..

[101] Mancuso M., Orsucci D., Angelini C., Bertini E., Carelli V., Comi G.P., Donati M.A., Federico A., Minetti C., Moggio M. (2015). Redefining phenotypes associated with mitochondrial DNA single deletion. J. Neurol..

[102] Wahbi K., Bougouin W., Behin A., Stojkovic T., Becane H.M., Jardel C., Berber N., Mochel F., Lombes A., Eymard B. (2015). Long-term cardiac prognosis and risk stratification in 260 adults presenting with mitochondrial diseases. Eur. Heart J..

[103] Ng Y.S., Grady J.P., Lax N.Z., Bourke J.P., Alston C.L., Hardy S.A., Falkous G., Schaefer A.G., Radunovic A., Mohiddin S.A. (2016). Sudden adult death syndrome in m. 3243A>G-related mitochondrial disease: An unrecognized clinical entity in young, asymptomatic adults. Eur. Heart J..

[104] Ng Y.S., Feeney C., Schaefer A.M., Holmes C.E., Hynd P., Alston C.L., Grady J.P., Roberts M., Maguire M., Bright A. (2016). Pseudo-obstruction, stroke, and mitochondrial dysfunction: A lethal combination. Ann. Neurol..

[105] Altmann J., Buchner B., Nadaj-Pakleza A., Schafer J., Jackson S., Lehmann D., Deschauer M., Kopajtich R., Lautenschlager R., Kuhn K.A. (2016). Expanded phenotypic spectrum of the m. 8344A>G “MERRF” mutation: Data from the German mitoNET registry. J. Neurol..

[106] Orsucci D., Angelini C., Bertini E., Carelli V., Comi G.P., Federico A., Minetti C., Moggio M., Mongini T., Santorelli F.M. (2017). Revisiting mitochondrial ocular myopathies: A study from the Italian Network. J. Neurol..

[107] Parikh S., Goldstein A., Karaa A., Koenig M.K., Anselm I., Brunel-Guitton C., Christodoulou J., Cohen B.H., Dimmock D., Enns G.M. (2017). Patient care standards for primary mitochondrial disease: A consensus statement from the Mitochondrial Medicine Society. Genet. Med..

[108] Garone C., Taylor R.W., Nascimento A., Poulton J., Fratter C., Domínguez-González C., Evans J.C., Loos M., Isohanni P., Suomalainen A. (2018). Retrospective natural history of thymidine kinase 2 deficiency. J. Med. Genet..

[109] Koene S., van Bon L., Bertini E., Jimenez-Moreno C., van der Giessen L., de Groot I., McFarland R., Parikh S., Rahman S., Wood M. (2018). Outcome measures for children with mitochondrial disease: Consensus recommendations for future studies from a Delphi-based international workshop. J. Inherit. Metab. Dis..

[110] Luo S., Valencia C.A., Zhang J., Lee N.-C., Slone J., Gui B., Wang X., Li Z., Dell S., Brown J. (2018). Biparental Inheritance of Mitochondrial DNA in Humans. Proc. Natl. Acad. Sci. USA.

[111] Ng Y.S., Martikainen M.H., Gorman G.S., Blain A., Bugiardini E., Bunting A., Schaefer A.M., Alston C.L., Blakely E.L., Sharma S. (2019). Pathogenic variants in MT-ATP6: A United Kingdom-based mitochondrial disease cohort study. Ann. Neurol..

[112] Parikh S., Karaa A., Goldstein A., Bertini E.S., Chinnery P.F., Christodoulou J., Cohen B.H., Davis R.L., Falk M.J., Fratter C. (2019). Diagnosis of ‘possible’ mitochondrial disease: An existential crisis. J. Med. Genet..

[113] Hikmat O., Naess K., Engvall M., Klingenberg C., Rasmussen M., Tallaksen C.M., Brodtkorb E., Ostergaard E., de Coo I.F.M., Pias-Peleteiro L. (2020). Simplifying the clinical classification of polymerase gamma (POLG) disease based on age of onset; studies using a cohort of 155 cases. J. Inherit. Metab. Dis..

[114] De Vries M.C., Brown D.A., Allen M.E., Bindoff L., Gorman G.S., Karaa A., Keshavan N., Lamperti C., McFarland R., Ng Y.S. (2020). Safety of drug use in patients with a primary mitochondrial disease: An international Delphi-based consensus. J. Inherit. Metab. Dis..

[115] Barca E., Long Y., Cooley V., Schoenaker R., Emmanuele V., DiMauro S., Cohen B.H., Karaa A., Vladutiu G.D., Haas R. (2020). Mitochondrial diseases in North America: An analysis of the NAMDC Registry. Neurol. Genet..

[116] de Laat P., Rodenburg R.R., Roeleveld N., Koene S., Smeitink J.A., Janssen M.C. (2020). Six-year prospective follow-up study in 151 carriers of the mitochondrial DNA 3243 A>G variant. J. Med. Genet..

[117] Montano V., Lopriore P., Gruosso F., Carelli V., Comi G.P., Filosto M., Lamperti C., Mongini T., Musumeci O., Servidei S. (2022). Primary mitochondrial myopathy: 12-month follow-up results of an Italian cohort. J. Neurol..

[118] Bender F., Timmann D., van de Warrenburg B.P., Adarmes-Gómez A.D., Bender B., Thieme A., Synofzik M., Schöls L. (2021). Natural History of Polymerase Gamma-Related Ataxia. Mov. Disord..

[119] Flickinger J., Fan J., Wellik A., Ganetzky R., Goldstein A., Muraresku C.C., Glanzman A.M., Ballance E., Leonhardt K., McCormick E.M. (2021). Development of a Mitochondrial Myopathy-Composite Assessment Tool. JCSM Clin. Rep..

[120] Lim A.Z., Ng Y.S., Blain A., Jiminez-Moreno C., Alston C.L., Nesbitt V., Simmons L., Santra S., Wassmer E., Blakely E.L. (2022). Natural History of Leigh Syndrome: A Study of Disease Burden and Progression. Ann. Neurol..

[121] Stenton S.L., Zou Y., Cheng H., Liu Z., Wang J., Shen D., Jin H., Ding C., Tang X., Sun S. (2022). Leigh Syndrome: A Study of 209 Patients at the Beijing Children’s Hospital. Ann. Neurol..

[122] Pizzamiglio C., Stefanetti R.J., McFarland R., Thomas N., Ransley G., Hugerth M., Grönberg A., Serrano S.S., Elmér E., Hanna M.G. (2025). Optimizing rare disorder trials: A phase 1a/1b randomized study of KL1333 in adults with mitochondrial disease. Brain.

[123] McCormick E.M., Keller K., Taylor J.P., Coffey A.J., Shen L., Krotoski D., Harding B., Gai X., Falk M.J., Zolkipli-Cunningham Z. (2023). Expert Panel Curation of 113 Primary Mitochondrial Disease Genes for the Leigh Syndrome Spectrum. Ann. Neurol..

[124] Ardissone A., Ferrera G., Lamperti C., Tiranti V., Ghezzi D., Moroni I., Lamantea E. (2023). Phenotyping mitochondrial DNA-related diseases in childhood: A cohort study of 150 patients. Eur. J. Neurol..

[125] Hikmat O., Naess K., Engvall M., Klingenberg C., Rasmussen M., Brodtkorb E., Ostergaard E., de Coo I., Pias-Peleteiro L., Isohanni P. (2024). Status epilepticus in POLG disease: A large multinational study. J. Neurol..

[126] Yu-Wai-Man P., Carelli V., Newman N.J., Silva M.J., Linden A., Van Stavern G., Szaflik J.P., Banik R., Lubiński W., Pemp B. (2024). Therapeutic benefit of idebenone in patients with Leber hereditary optic neuropathy: The LEROS nonrandomized controlled trial. Cell Rep. Med..

[127] Domínguez-González C., Chiang C., Colson A.O., Rebollo Mesa I., Baixauli E., Quan J., VanMeter S., Hirano M. (2025). Pyrimidine Nucleos(t)ide Therapy in Patients with Thymidine Kinase 2 Deficiency: A Multicenter Retrospective Chart Review Study. Neurology.

[128] Ivaniuk A., Anselm I.A., Bowen A., Cohen B.H., Eminoglu F.T., Estrella J., Gallagher R.C., Ganetzky R.D., Gannon J., Gorman G.S. (2025). Characterization of Factors Associated with Death in Deceased Patients with Mitochondrial Disorders: A Multicenter Cross-Sectional Survey. Neurology.

[129] Alves C., Rossi-Espagnet M.C., Perez F., Manteghinejad A., Peterson J.T., Ganetzky R., Napolitano A., Grassi F., George-Sankoh I., Yildiz H. (2025). Single Large-Scale Mitochondrial Deletion Syndromes: Neuroimaging Phenotypes and Longitudinal Progression in Pediatric Patients. AJNR Am. J. Neuroradiol..

[130] Merkevicius K., Smirnov D., Schlieben L.D., Ganetzky R., Feichtinger R.G., Jiang H., Fang F., Ebihara T., Murayama K., Ferrera G. (2025). The genotypic and phenotypic landscape of PDHA1-related pyruvate dehydrogenase complex deficiency. Brain.

[131] Mancuso M., Bellusci M., Carelli V., de Coo I., Diodato D., Distelmaier F., Hikmat O., Hirano M., Horvath R., Karaa A. (2026). Diagnostic Criteria and Management of MELAS and Stroke-Like Episodes: Consensus-Based Statements. Eur. J. Neurol..

[132] Hyslop L.A., Blakely E.L., Aushev M., Marley J., Takeda Y., Pyle A., Moody E., Feeney C., Dutton J., Shaw C. (2025). Mitochondrial Donation and Preimplantation Genetic Testing for mtDNA Disease. N. Engl. J. Med..

[133] McFarland R., Hyslop L.A., Feeney C., Pillai R.N., Blakely E.L., Moody E., Prior M., Devlin A., Taylor R.W., Herbert M. (2025). Mitochondrial Donation in a Reproductive Care Pathway for mtDNA Disease. N. Engl. J. Med..

[134] Lopriore P., Ünlütürk Z., Klopstock T., Karaa A., Rouzier C., Domínguez-González C., Lamperti C., Mancuso M., Cecchi G., Montano V. (2026). Clinical and Genotypic Spectrum of Twinkle-Related Disorders: Insights From a Multinational Cohort Study. Neurology.

[135] Rahman S., Blok R.B., Dahl H.H., Danks D.M., Kirby D.M., Chow C.W., Christodoulou J., Thorburn D.R. (1996). Leigh syndrome: Clinical features and biochemical and DNA abnormalities. Ann. Neurol..

[136] Leigh D. (1951). Subacute necrotizing encephalomyelopathy in an infant. J. Neurol. Neurosurg. Psychiatry.

[137] Watson-Fargie T., Marshall V., Fullerton N.E., Leach V., Pilz D., Hemingbrough C.V.Y., Hopton S., Taylor R.W., Ng Y.S., Schaefer A. (2024). Leigh syndrome: An adult presentation of a paediatric disease. Pract. Neurol..

[138] Hong C.M., Na J.H., Park S., Lee Y.M. (2020). Clinical Characteristics of Early-Onset and Late-Onset Leigh Syndrome. Front. Neurol..

[139] Rahman S. (2023). Leigh syndrome. Handb. Clin. Neurol..

[140] Ardissone A., Bruno C., Diodato D., Donati A., Ghezzi D., Lamantea E., Lamperti C., Mancuso M., Martinelli D., Primiano G. (2021). Clinical, imaging, biochemical and molecular features in Leigh syndrome: A study from the Italian network of mitochondrial diseases. Orphanet J. Rare Dis..

[141] Hayhurst H., de Coo I.F.M., Piekutowska-Abramczuk D., Alston C.L., Sharma S., Thompson K., Rius R., He L., Hopton S., Ploski R. (2019). Leigh syndrome caused by mutations in MTFMT is associated with a better prognosis. Ann. Clin. Transl. Neurol..

[142] Kristensen E., Mathisen L., Berland S., Klingenberg C., Brodtkorb E., Rasmussen M., Tangeraas T., Bliksrud Y.T., Rahman S., Bindoff L.A. (2024). Epidemiology and natural history of POLG disease in Norway: A nationwide cohort study. Ann. Clin. Transl. Neurol..

[143] Rötig A., Gaignard P., Barcia G., Assouline Z., Berat C.M., Barth M., Damaj L., Laborde N., Abi-Warde M.T., Chabrol B. (2024). Distinct Clinical Courses and Shortened Lifespans in Childhood-Onset DNA Polymerase Gamma Deficiency. Neurol. Genet..

[144] Smith L.A., Olkhova E.A., Lax N.Z., Ng Y.S., Taylor R.W., Gorman G.S., Erskine D., McFarland R. (2025). Delineating the mechanisms of cerebellar degeneration in paediatric and adult primary mitochondrial disease. Acta Neuropathol..

[145] Moe A., Fry C., Baker M.R., Ng Y.S. (2026). A 38-year-old man with unsteadiness and difficulty walking. Pract. Neurol..

[146] Cohen B.H., Chinnery P.F., Copeland W.C., Adam M.P., Bick S., Mirzaa G.M., Pagon R.A., Wallace S.E., Amemiya A. (1993). POLG-Related Disorders. GeneReviews(^®^).

[147] Patel K.P., O’Brien T.W., Subramony S.H., Shuster J., Stacpoole P.W. (2012). The spectrum of pyruvate dehydrogenase complex deficiency: Clinical, biochemical and genetic features in 371 patients. Mol. Genet. Metab..

[148] Savvidou A., Sofou K., Eklund E.A., Aronsson J., Darin N. (2024). Manifestations of X-linked pyruvate dehydrogenase complex deficiency in female PDHA1 carriers. Eur. J. Neurol..

[149] Betesh-Abay B., Shany E., Staretz-Chacham O., Shelef I., Azab A.N. (2026). Pyruvate Dehydrogenase Complex Deficiency: A Review of Treatments and Case Series. Int. J. Mol. Sci..

[150] Orsucci D., Caldarazzo Ienco E., Lopriore P., Mancuso M. (2025). Disease registries and rare disorders: The virtuous example of mitochondrial medicine. Exp. Neurol..

[151] Rosales X.Q., Thompson J.L.P., Haas R., Van Hove J.L.K., Karaa A., Krotoski D., Engelstad K., Buchsbaum R., DiMauro S., Hirano M. (2020). The North American mitochondrial disease registry. J. Transl. Genet. Genom..

[152] Shi Y., Chen G., Sun D., Hu C., Liu Z., Shen D., Wang J., Song T., Zhang W., Li J. (2022). Phenotypes and genotypes of mitochondrial diseases with mtDNA variations in Chinese children: A multi-center study. Mitochondrion.

[153] Stenton S., Shimura M., Piekutowska-Abramczuk D., Freisinger P., Distelmaier F., Mayr J., Makowski C., Büchner B., Alhaddad B., Alston C. (2021). Diagnosing pediatric mitochondrial disease: Lessons from 2,000 exomes. medRxiv.

[154] Lynch D.R., Goldsberry A., Rummey C., Farmer J., Boesch S., Delatycki M.B., Giunti P., Hoyle J.C., Mariotti C., Mathews K.D. (2024). Propensity matched comparison of omaveloxolone treatment to Friedreich ataxia natural history data. Ann. Clin. Transl. Neurol..

[155] Leigh Syndrome International Consortium. https://leighsyndrome.org/the-leigh-syndrome-consortium/.

[156] MacMullen L.E., George-Sankoh I., Stanley K., McCormick E.M., Muraresku C.C., Goldstein A., Zolkipli-Cunningham Z., Falk M.J. (2024). Bridging the clinical-research gap: Harnessing an electronic data capture, integration, and visualization platform to systematically assess prospective patient-reported outcomes in mitochondrial medicine. Mol. Genet. Metab..

[157] Lim A.Z., Jones D.M., Bates M.G.D., Schaefer A.M., O’Sullivan J., Feeney C., Farrugia M.E., Bourke J.P., Turnbull D.M., Gorman G.S. (2021). Risk of cardiac manifestations in adult mitochondrial disease caused by nuclear genetic defects. Open Heart.

[158] Savvatis K., Vissing C.R., Klouvi L., Florian A., Rahman M., Béhin A., Fayssoil A., Masingue M., Stojkovic T., Bécane H.M. (2022). Cardiac Outcomes in Adults with Mitochondrial Diseases. J. Am. Coll. Cardiol..

[159] Nikhanj A., Bautista J., Siddiqi Z.A., Phan C.L., Oudit G.Y. (2022). Low Prevalence of Cardiomyopathy in Patients with Mitochondrial Disease and Neurological Manifestations. J. Cardiovasc. Dev. Dis..

[160] Weiner J.G., Lambert A.N., Thurm C., Hall M., Soslow J.H., Reimschisel T.E., Bearl D.W., Dodd D.A., Feingold B., Godown J. (2020). Heart Transplantation in Children with Mitochondrial Disease. J. Pediatr..

[161] Wong S.W., Fung C.W., Tomlin F., Stojanovic J. (2025). Kidney transplantation in mitochondrial diseases: A systematic review. Pediatr. Nephrol..

[162] Parikh S., Karaa A., Goldstein A., Ng Y.S., Gorman G., Feigenbaum A., Christodoulou J., Haas R., Tarnopolsky M., Cohen B.K. (2016). Solid organ transplantation in primary mitochondrial disease: Proceed with caution. Mol. Genet. Metab..

[163] Vara R., Pinon M., Fratter C., Hegarty R., Hadzic N. (2023). Hepatic presentations of mitochondrial DNA depletion syndrome in children: A single tertiary liver centre experience. J. Inherit. Metab. Dis..

[164] Shimura M., Kuranobu N., Ogawa-Tominaga M., Akiyama N., Sugiyama Y., Ebihara T., Fushimi T., Ichimoto K., Matsunaga A., Tsuruoka T. (2020). Clinical and molecular basis of hepatocerebral mitochondrial DNA depletion syndrome in Japan: Evaluation of outcomes after liver transplantation. Orphanet J. Rare Dis..

[165] Keshavan N., Rahman S. (2018). Natural history of mitochondrial disorders: A systematic review. Essays Biochem..

[166] Domínguez-González C., Madruga-Garrido M., Hirano M., Martí I., Martín M.A., Munell F., Nascimento A., Olivé M., Quan J., Sardina M.D. (2021). Collaborative model for diagnosis and treatment of very rare diseases: Experience in Spain with thymidine kinase 2 deficiency. Orphanet J. Rare Dis..

[167] Domínguez-González C., Hernández-Laín A., Rivas E., Hernández-Voth A., Sayas Catalán J., Fernández-Torrón R., Fuiza-Luces C., García García J., Morís G., Olivé M. (2019). Late-onset thymidine kinase 2 deficiency: A review of 18 cases. Orphanet J. Rare Dis..

[168] Ceballos F., Serrano-Lorenzo P., Bermejo-Guerrero L., Blázquez A., Quesada-Espinosa J.F., Amigo J., Minguez P., Ayuso C., García-Arumí E., Muelas N. (2024). Clinical and Genetic Analysis of Patients with TK2 Deficiency. Neurol. Genet..

[169] Neugebauer J., Reinson K., Bellusci M., Park J.H., Hikmat O., Bertini E., Schiff M., Rahman S. (2025). Current global vitamin and cofactor prescribing practices for primary mitochondrial diseases: Results of a European reference network survey. J. Inherit. Metab. Dis..

[170] Stefanetti R.J., Ng Y.S., Errington L., Blain A.P., McFarland R., Gorman G.S. (2022). l-Arginine in Mitochondrial Encephalopathy, Lactic Acidosis, and Stroke-like Episodes: A Systematic Review. Neurology.

[171] Ng Y.S., Bindoff L.A., Gorman G.S., Horvath R., Klopstock T., Mancuso M., Martikainen M.H., McFarland R., Nesbitt V., Pitceathly R.D.S. (2019). Consensus-based statements for the management of mitochondrial stroke-like episodes. Wellcome Open Res..

[172] van Everdingen J.A.M., Pott J.W.R., Bauer N.J.C., Krijnen A.M., Lushchyk T., Wubbels R.J. (2022). Clinical outcomes of treatment with idebenone in Leber’s hereditary optic neuropathy in The Netherlands: A national cohort study. Acta Ophthalmol..

[173] Yu-Wai-Man P., Newman N.J., Carelli V., Moster M.L., Biousse V., Sadun A.A., Klopstock T., Vignal-Clermont C., Sergott R.C., Rudolph G. (2020). Bilateral visual improvement with unilateral gene therapy injection for Leber hereditary optic neuropathy. Sci. Transl. Med..

[174] Newman N.J., Yu-Wai-Man P., Carelli V., Moster M.L., Biousse V., Vignal-Clermont C., Sergott R.C., Klopstock T., Sadun A.A., Barboni P. (2021). Efficacy and Safety of Intravitreal Gene Therapy for Leber Hereditary Optic Neuropathy Treated within 6 Months of Disease Onset. Ophthalmology.

[175] Yu-Wai-Man P., Newman N.J., Biousse V., Carelli V., Moster M.L., Vignal-Clermont C., Klopstock T., Sadun A.A., Sergott R.C., Hage R. (2025). Five-Year Outcomes of Lenadogene Nolparvovec Gene Therapy in Leber Hereditary Optic Neuropathy. JAMA Ophthalmol..

[176] Newman N.J., Yu-Wai-Man P., Subramanian P.S., Moster M.L., Wang A.G., Donahue S.P., Leroy B.P., Carelli V., Biousse V., Vignal-Clermont C. (2023). Randomized trial of bilateral gene therapy injection for m. 11778G>A MT-ND4 Leber optic neuropathy. Brain.

[177] Gorman G.S., Elson J.L., Newman J., Payne B., McFarland R., Newton J.L., Turnbull D.M. (2015). Perceived fatigue is highly prevalent and debilitating in patients with mitochondrial disease. Neuromuscul. Disord..

[178] Jeppesen T.D., Madsen K.L., Poulsen N.S., Løkken N., Vissing J. (2021). Exercise Testing, Physical Training and Fatigue in Patients with Mitochondrial Myopathy Related to mtDNA Mutations. J. Clin. Med..

[179] Parikh S., Galioto R., Lapin B., Haas R., Hirano M., Koenig M.K., Saneto R.P., Zolkipli-Cunningham Z., Goldstein A., Karaa A. (2019). Fatigue in primary genetic mitochondrial disease: No rest for the weary. Neuromuscul. Disord..

[180] Clifford S., Stefanetti R.J., Bahar R., Hansson M.J., Gorman G.S., Karaa A. (2025). Qualitative study of fatigue in adults with primary mitochondrial disease: Development of the PROMIS Fatigue Mitochondrial Disease Short Form. Mol. Genet. Metab..

[181] Klein I.L., Verhaak C.M., Smeitink J.A.M., de Laat P., Janssen M.C.H., Custers J.A.E. (2022). Identifying trajectories of fatigue in patients with primary mitochondrial disease due to the m. 3243A>G variant. J. Inherit. Metab. Dis..

[182] Stefanetti R.J., Charman S.J., Newman J., Hallsworth K., Blain A.P., Ng Y.S., Gorman G.S. (2025). Outcomes misaligned in mitochondrial encephalomyopathy, lactic acidosis and stroke-like episodes (MELAS): Implications for trial design. Brain Commun..

[183] Russell O.M., Gorman G.S., Lightowlers R.N., Turnbull D.M. (2020). Mitochondrial Diseases: Hope for the Future. Cell.

[184] Abouhajar A., Alcock L., Bigirumurame T., Bradley P., Brown L., Campbell I., Del Din S., Faitg J., Falkous G., Gorman G.S. (2022). Acipimox in Mitochondrial Myopathy (AIMM): Study protocol for a randomised, double-blinded, placebo-controlled, adaptive design trial of the efficacy of acipimox in adult patients with mitochondrial myopathy. Trials.

[185] van Tienen F.H.J., Hoeijmakers J.G.J., van der Leij C., Timmer E., Wanders N., Lindsey P.J., Yi F., Lin F., Kortekaas S.P.M., Roelofs H. (2025). Intra-arterial transplantation of autologous mesoangioblasts in m. 3243A>G mutation carriers is safe: First phase 1/2 human clinical study. Mol. Ther..

[186] Steele H., Gomez-Duran A., Pyle A., Hopton S., Newman J., Stefanetti R.J., Charman S.J., Parikh J.D., He L., Viscomi C. (2020). Metabolic effects of bezafibrate in mitochondrial disease. EMBO Mol. Med..

[187] Pekeles H., Berrahmoune S., Dassi C., Cheung A.C.T., Gagnon T., Waters P.J., Eberhard R., Buhas D., Myers K.A. (2024). Safety and efficacy of deoxycytidine/deoxythymidine combination therapy in POLG-related disorders: 6-month interim results of an open-label, single arm, phase 2 trial. EClinicalMedicine.

[188] Karaa A., Haas R., Goldstein A., Vockley J., Weaver W.D., Cohen B.H. (2018). Randomized dose-escalation trial of elamipretide in adults with primary mitochondrial myopathy. Neurology.

[189] Karaa A., Haas R., Goldstein A., Vockley J., Cohen B.H. (2020). A randomized crossover trial of elamipretide in adults with primary mitochondrial myopathy. J. Cachexia Sarcopenia Muscle.

[190] Karaa A., Bertini E., Carelli V., Cohen B.H., Enns G.M., Falk M.J., Goldstein A., Gorman G.S., Haas R., Hirano M. (2023). Efficacy and Safety of Elamipretide in Individuals with Primary Mitochondrial Myopathy: The MMPOWER-3 Randomized Clinical Trial. Neurology.

[191] Thompson W.R., Manuel R., Abbruscato A., Carr J., Campbell J., Hornby B., Vaz F.M., Vernon H.J. (2024). Long-term efficacy and safety of elamipretide in patients with Barth syndrome: 168-week open-label extension results of TAZPOWER. Genet. Med..

[192] Karanjia R., Sadun A.A. (2024). Elamipretide Topical Ophthalmic Solution for the Treatment of Subjects with Leber Hereditary Optic Neuropathy: A Randomized Trial. Ophthalmology.

[193] Guerrero-Molina M.P., Morales-Conejo M., Delmiro A., Morán M., Domínguez-González C., Arranz-Canales E., Ramos-González A., Arenas J., Martín M.A., de la Aleja J.G. (2023). High-dose oral glutamine supplementation reduces elevated glutamate levels in cerebrospinal fluid in patients with mitochondrial encephalomyopathy, lactic acidosis and stroke-like episodes syndrome. Eur. J. Neurol..

[194] Wong T.S., Man E., Wong I.C.K., Li S.X., Zhi H., Yeung K.M.C., Yip K.Y., Kwong A.K.Y., Belaramani K.M., Wong S.N. (2025). Open-label pilot study using hydroxytyrosol as dietary supplements in patients with mitochondrial diseases. Orphanet J. Rare Dis..

[195] Koga Y., Povalko N., Inoue E., Nakamura H., Ishii A., Suzuki Y., Yoneda M., Kanda F., Kubota M., Okada H. (2018). Therapeutic regimen of L-arginine for MELAS: 9-year, prospective, multicenter, clinical research. J. Neurol..

[196] Al Jasmi F., Al Zaabi N., Al-Thihli K., Al Teneiji A.M., Hertecant J., El-Hattab A.W. (2020). Endothelial Dysfunction and the Effect of Arginine and Citrulline Supplementation in Children and Adolescents with Mitochondrial Diseases. J. Cent. Nerv. Syst. Dis..

[197] Vignal C., Uretsky S., Fitoussi S., Galy A., Blouin L., Girmens J.F., Bidot S., Thomasson N., Bouquet C., Valero S. (2018). Safety of rAAV2/2-ND4 Gene Therapy for Leber Hereditary Optic Neuropathy. Ophthalmology.

[198] Newman N.J., Yu-Wai-Man P., Carelli V., Biousse V., Moster M.L., Vignal-Clermont C., Sergott R.C., Klopstock T., Sadun A.A., Girmens J.F. (2021). Intravitreal Gene Therapy vs. Natural History in Patients with Leber Hereditary Optic Neuropathy Carrying the m. 11778G>A ND4 Mutation: Systematic Review and Indirect Comparison. Front. Neurol..

[199] Houghton D., Ng Y.S., Jackson M.A., Stefanetti R., Hynd P., Mac Aogáin M., Stewart C.J., Lamb C.A., Bright A., Feeney C. (2022). Phase II Feasibility Study of the Efficacy, Tolerability, and Impact on the Gut Microbiome of a Low-Residue (Fiber) Diet in Adult Patients with Mitochondrial Disease. Gastro Hep Adv..

[200] Stefanetti R.J., Pizzamiglio C., Blain A.P., Russell O.M., Alcock L., Hudson G., Newman J., Thomas N., Warren C., Su H. (2026). Mavodelpar in patients with primary mitochondrial myopathy: A phase 1 trial. Sci. Rep..

[201] Pirinen E., Auranen M., Khan N.A., Brilhante V., Urho N., Pessia A., Hakkarainen A., Kuula J., Heinonen U., Schmidt M.S. (2020). Niacin Cures Systemic NAD(+) Deficiency and Improves Muscle Performance in Adult-Onset Mitochondrial Myopathy. Cell Metab..

[202] Madsen K.L., Buch A.E., Cohen B.H., Falk M.J., Goldsberry A., Goldstein A., Karaa A., Koenig M.K., Muraresku C.C., Meyer C. (2020). Safety and efficacy of omaveloxolone in patients with mitochondrial myopathy: MOTOR trial. Neurology.

[203] Lynch D.R., Chin M.P., Delatycki M.B., Subramony S.H., Corti M., Hoyle J.C., Boesch S., Nachbauer W., Mariotti C., Mathews K.D. (2021). Safety and Efficacy of Omaveloxolone in Friedreich Ataxia (MOXIe Study). Ann. Neurol..

[204] Liu H.L., Yuan J.J., Zhang Y., Tian Z., Li X., Wang D., Du Y.Y., Song L., Li B. (2020). Factors associated with rapid improvement in visual acuity in patients with Leber’s hereditary optic neuropathy after gene therapy. Acta Ophthalmol..

[205] Storgaard J.H., Løkken N., Madsen K.L., Voermans N.C., Laforêt P., Nadaj-Pakleza A., Tard C., van Hall G., Vissing J., Ørngreen M.C. (2022). No effect of resveratrol on fatty acid oxidation or exercise capacity in patients with fatty acid oxidation disorders: A randomized clinical cross-over trial. J. Inherit. Metab. Dis..

[206] Feuer W.J., Schiffman J.C., Davis J.L., Porciatti V., Gonzalez P., Koilkonda R.D., Yuan H., Lalwani A., Lam B.L., Guy J. (2016). Gene Therapy for Leber Hereditary Optic Neuropathy: Initial Results. Ophthalmology.

[207] Abe Y., Hamasaki T., Natsume J., Mogami Y., Murayama K., Shiraishi H., Abe Y., Kumada S., Tanaka R., Ihara K. (2025). Efficacy and Safety of 5-Aminolevulinic Acid Hydrochloride Combined with Sodium Ferrous Citrate in Pediatric Patients with Leigh Syndrome and Central Nervous System Disorders: An Initial Exploratory Trial with a Double-Blind Placebo-Controlled Period, Followed by an Open-Label Period and a Subsequent Long-Term Administration Study. Life.

[208] Smeitink J., van Es J., Bosman B., Janssen M.C.H., Klopstock T., Gorman G., Vissing J., Ruiterkamp G., Edgar C.J., Abbink E.J. (2025). Phase 2b program with sonlicromanol in patients with mitochondrial disease due to m. 3243A>G mutation. Brain.

[209] Janssen M.C.H., Koene S., de Laat P., Hemelaar P., Pickkers P., Spaans E., Beukema R., Beyrath J., Groothuis J., Verhaak C. (2019). The KHENERGY Study: Safety and Efficacy of KH176 in Mitochondrial m. 3243A>G Spectrum Disorders. Clin. Pharmacol. Ther..

[210] Bangel K.A., Lim A.Z., Blain A., Ng Y.S., Winder A., Bulmer J., Gorman G., Baker M., McFarland R. (2024). TRANscranial direct current stimulation for FOcal Refractory epilepsy in mitochondrial disease (TRANSFORM): Delayed-start, randomised, double-blinded, placebo-controlled study. BMC Neurol..

[211] Profeta V., McIntyre K., Wells M., Park C., Lynch D.R. (2023). Omaveloxolone: An activator of Nrf2 for the treatment of Friedreich ataxia. Expert. Opin. Investig. Drugs.

[212] Lynch D.R., Chin M.P., Boesch S., Delatycki M.B., Giunti P., Goldsberry A., Hoyle J.C., Mariotti C., Mathews K.D., Nachbauer W. (2023). Efficacy of Omaveloxolone in Friedreich’s Ataxia: Delayed-Start Analysis of the MOXIe Extension. Mov. Disord..

[213] Jain I.H., Zazzeron L., Goli R., Alexa K., Schatzman-Bone S., Dhillon H., Goldberger O., Peng J., Shalem O., Sanjana N.E. (2016). Hypoxia as a therapy for mitochondrial disease. Science.

[214] Ferrari M., Jain I.H., Goldberger O., Rezoagli E., Thoonen R., Cheng K.H., Sosnovik D.E., Scherrer-Crosbie M., Mootha V.K., Zapol W.M. (2017). Hypoxia treatment reverses neurodegenerative disease in a mouse model of Leigh syndrome. Proc. Natl. Acad. Sci. USA.

[215] Martinelli D., Catteruccia M., Piemonte F., Pastore A., Tozzi G., Dionisi-Vici C., Pontrelli G., Corsetti T., Livadiotti S., Kheifets V. (2012). EPI-743 reverses the progression of the pediatric mitochondrial disease—Genetically defined Leigh Syndrome. Mol. Genet. Metab..

[216] Zink A., Dai D.F., Wittich A., Henke M.T., Pedrotti G., Heiduschka S., Santamaria G., Pentimalli T.M., Brueser C., Notopoulou S. (2026). Pluripotent stem-cell-based screening uncovers sildenafil as a mitochondrial disease therapy. Cell.

[217] Jacoby E., Blumkin M., Anikster Y., Varda-Bloom N., Pansheen J., Bar Yoseph O., Gruber N., Lahav E., Besser M.J., Schachter J. (2018). First-in-Human Mitochondrial Augmentation of Hematopoietic Stem Cells in Pearson Syndrome. Blood.

[218] Jacoby E., Bar-Yosef O., Gruber N., Lahav E., Varda-Bloom N., Bolkier Y., Bar D., Blumkin M.B., Barak S., Eisenstein E. (2022). Mitochondrial augmentation of hematopoietic stem cells in children with single large-scale mitochondrial DNA deletion syndromes. Sci. Transl. Med..

[219] Stendel C., Neuhofer C., Floride E., Yuqing S., Ganetzky R.D., Park J., Freisinger P., Kornblum C., Kleinle S., Schöls L. (2020). Delineating MT-ATP6-associated disease: From isolated neuropathy to early onset neurodegeneration. Neurol. Genet..

[220] Yu-Wai-Man P., Chinnery P.F., Adam M.P., Bick S., Mirzaa G.M., Pagon R.A., Wallace S.E., Amemiya A. (1993). Leber Hereditary Optic Neuropathy. GeneReviews(^®^).

[221] Steffann J. (2025). Advances in Preventing Transmission of Mitochondrial DNA Diseases. N. Engl. J. Med..

[222] Smeets H.J.M., Sallevelt S., Herbert M. (2023). Reproductive options in mitochondrial disease. Handb. Clin. Neurol..

[223] Craven L., Murphy J., Turnbull D.M., Taylor R.W., Gorman G.S., McFarland R. (2018). Scientific and Ethical Issues in Mitochondrial Donation. New Bioeth..

[224] Herbrand C. (2022). Silences, omissions and oversimplification? The UK debate on mitochondrial donation. Reprod. Biomed. Soc. Online.

[225] Castelluccio N., Spath K., Li D., De Coo I.F.M., Butterworth L., Wells D., Mertes H., Poulton J., Heindryckx B. (2025). Genetic and reproductive strategies to prevent mitochondrial diseases. Human. Reprod. Update.

[226] Ilg W., Milne S., Schmitz-Hübsch T., Alcock L., Beichert L., Bertini E., Mohamed Ibrahim N., Dawes H., Gomez C.M., Hanagasi H. (2024). Quantitative Gait and Balance Outcomes for Ataxia Trials: Consensus Recommendations by the Ataxia Global Initiative Working Group on Digital-Motor Biomarkers. Cerebellum.

[227] Servais L., Eggenspieler D., Poleur M., Grelet M., Muntoni F., Strijbos P., Annoussamy M. (2023). First regulatory qualification of a digital primary endpoint to measure treatment efficacy in DMD. Nat. Med..

[228] Pfeffer G., Horvath R., Klopstock T., Mootha V.K., Suomalainen A., Koene S., Hirano M., Zeviani M., Bindoff L.A., Yu-Wai-Man P. (2013). New treatments for mitochondrial disease—No time to drop our standards. Nat. Rev. Neurol..

[229] Medrano P., Banderas B., Brimmer M., Settel L., Berger S., Shields A., Goldstein A., Karaa A., Larson A., Parikh S. (2025). Signs, symptoms, and health-related quality of life in MELAS: Measuring what’s important from the patient and clinician perspectives. J. Patient Rep. Outcomes.

[230] Jimenez-Moreno A.C., van Overbeeke E., Pinto C.A., Smith I., Sharpe J., Ormrod J., Whichello C., de Bekker-Grob E.W., Bullok K., Levitan B. (2021). Patient Preferences in Rare Diseases: A Qualitative Study in Neuromuscular Disorders to Inform a Quantitative Preference Study. Patient.

[231] Park J.J.H., Siden E., Zoratti M.J., Dron L., Harari O., Singer J., Lester R.T., Thorlund K., Mills E.J. (2019). Systematic review of basket trials, umbrella trials, and platform trials: A landscape analysis of master protocols. Trials.

[232] Devanarayan V., Donohue M.C., Sperling R.A., Johnson K.A., Ye Y., Charil A., Doherty T., Tian L., Raman R., Aisen P.S. (2025). Multimodal prognostic modeling of individual cognitive trajectories to enhance trial efficiency in preclinical Alzheimer’s disease. Alzheimers Dement..

[233] Villalón-García I., Álvarez-Córdoba M., Suárez-Rivero J.M., Povea-Cabello S., Talaverón-Rey M., Suárez-Carrillo A., Munuera-Cabeza M., Sánchez-Alcázar J.A. (2020). Precision Medicine in Rare Diseases. Diseases.

[234] Keshavan N., Minczuk M., Viscomi C., Rahman S. (2024). Gene therapy for mitochondrial disorders. J. Inherit. Metab. Dis..

